# Mutations in the bone morphogenetic protein signaling pathway sensitize zebrafish and humans to ethanol-induced jaw malformations

**DOI:** 10.1242/dmm.052223

**Published:** 2025-04-08

**Authors:** John R. Klem, Tae-Hwi Schwantes-An, Marco Abreu, Michael Suttie, Raèden Gray, Hieu D. L. Vo, Grace Conley, Tatiana M. Foroud, Leah Wetherill, Collaborative Initiative on Fetal Alcohol Spectrum Disorders (CIFASD), C. Ben Lovely

**Affiliations:** ^1^ University of Louisville School of Medicine, Department of Biochemistry and Molecular Genetics, Alcohol Research Center, Louisville, KY 40202, USA; ^2^ Department of Medical and Molecular Genetics, Indiana University School of Medicine, Indianapolis, IN 40202, USA; ^3^ Nuffield Department of Women's and Reproductive Health, University of Oxford, Oxford OX3 9DU, UK; ^4^ Big Data Institute, University of Oxford, Oxford OX3 7LF, UK; ^5^ https://cifasd.org/

**Keywords:** Fetal alcohol spectrum disorders, Zebrafish, Alcohol, Genetics, Endoderm, Jaw development

## Abstract

Fetal alcohol spectrum disorders (FASD) describe ethanol-induced developmental defects including craniofacial malformations. While ethanol-sensitive genetic mutations contribute to facial malformations, the impacted cellular mechanisms remain unknown. Signaling via bone morphogenetic protein (Bmp) is a key regulatory step of epithelial morphogenesis driving facial development, providing a possible ethanol-sensitive mechanism. We found that zebrafish carrying mutants for Bmp signaling components are ethanol-sensitive and affect anterior pharyngeal endoderm shape and gene expression, indicating that ethanol-induced malformations of the anterior pharyngeal endoderm cause facial malformations. By integrating FASD patient data, we provide the first evidence that variants of the human Bmp receptor gene *BMPR1B* associate with ethanol-related differences in jaw volume. Our results show that ethanol exposure disrupts proper morphogenesis of, and tissue interactions between, facial epithelia that mirror overall viscerocranial shape changes and are predictive for Bmp−ethanol associations in human jaw development. Our data provide a mechanistic paradigm linking ethanol to disrupted epithelial cell behaviors that underlie facial defects in FASD.

## Teaser

**Summary:** We apply a unique combination of zebrafish-based approaches, and human genetics and facial dysmorphology analyses to resolve the cellular mechanisms that are driven by the ethanol-sensitive Bmp pathway.

## INTRODUCTION

Ethanol is the most common environmental risk factor for congenital anomalies, with fetal alcohol spectrum disorders (FASD) describing all ethanol-induced birth defects. Global estimated incidence rates of FASD are 0.77%, although subpopulations of FASD can be much higher, ranging from 2-5% in the USA and to nearly 30% of individuals in some parts of the world with higher incidences of binge drinking (May et al., 2018; Popova et al., 2019). However, these numbers may be underestimates as nearly half of all pregnancies in the USA are unplanned, and many pediatricians fail to recognize FASD (Finer and Zolna, 2016; Rojmahamongkol et al., 2015). Highly variable, multiple phenotypes present with FASD include structural malformations to the brain and face (Lovely, 2020). At the most-severe end of the FASD spectrum is fetal alcohol syndrome (FAS), which frequently presents as craniofacial defects including jaw hypoplasia (Blanck-Lubarsch et al., 2020). However, increasing data indicate that prenatal alcohol exposure (PAE) results in craniofacial defects in the absence of a diagnosis of FAS (Muggli et al., 2017; Suttie et al., 2013). Multiple factors contribute to the impact of PAE, in particular genetic predisposition (Lovely, 2020). To date, several ethanol-sensitizing alleles in zebrafish, mouse and human have been linked to increased cell death, holoprosencephaly, oral clefting, disruption to axonal projections, and broad neural and eye defects (Boyles et al., 2010; Hong and Krauss, 2012; McCarthy et al., 2013; Swartz et al., 2014; Zhang et al., 2013). Despite these growing insights into the genetic components contributing to risk for FASD, we lack mechanistic insights into ethanol-sensitive gene function and associated cellular mechanisms during development that underlie FASD (Lovely, 2020).

Increasing evidence from several vertebrates including humans shows that genetic factors modulate developmental ethanol sensitivity (Lovely, 2020). Studying FASD in humans remains challenging due to the complex interplay of genetic background with − often incompletely documented − ethanol timing and dosage. Genetically tractable model organisms, such as zebrafish, have been essential in improving our understanding of the genetic loci behind the variability in FASD (Fernandes and Lovely, 2021). The zebrafish is well-suited for studying ethanol-sensitizing genetics because of its genetic tractability, high fecundity, external fertilization, embryo transparency and rapid development (McCarthy et al., 2013; Swartz et al., 2014, 2020). We have previously used both a candidate-based unbiased forward-genetic screen-based approach to identify ethanol-sensitive mutations (McCarthy et al., 2013; Swartz et al., 2014, 2020) and these approaches have proven successful in predicting human gene−ethanol interactions (McCarthy et al., 2013). However, despite the increasing number of identified ethanol-sensitive loci, we lack the conceptual and mechanistic understanding of how these gene−ethanol interactions affect the diverse cell types and cellular behaviors that underlie craniofacial development.

Previous work has established that both the genetic pathways required in, and the cellular events for, development of the craniofacial skeleton are deeply conserved between zebrafish and mammals (Knight and Schilling, 2006; Medeiros and Crump, 2012; Murillo-Rincón and Kaucka, 2020). Cranial neural crest cells (CNCC) give rise to the majority of the craniofacial skeleton and migrate from the dorsal neural tube to populate progenitor structures called the pharyngeal arches (Knight and Schilling, 2006; Medeiros and Crump, 2012; Murillo-Rincón and Kaucka, 2020). Concurrent with cranial neural crest cell migration, the pharyngeal endoderm undergoes its own cellular rearrangements and tissue movements to form a midline epithelial sheet with lateral protrusions called ‘pouches’. Proper morphogenesis of the pharyngeal endoderm is critical for craniofacial development, in particular of the jaw (Balczerski et al., 2012; Couly et al., 2002; Crump et al., 2004; Haworth et al., 2004, 2007; Lovely et al., 2016). Work in zebrafish has shown that several genetic pathways regulate endodermal morphogenesis (Balczerski et al., 2012; Choe et al., 2013; Choe and Crump, 2014; Crump et al., 2004; Hu et al., 2018; Li et al., 2019; Lovely et al., 2016). One such pathway is the bone morphogenetic protein (Bmp) signaling pathway. Comprising over 60 pathway components with different spatio-temporal expression and activity, active Bmp signaling is initiated with heterodimer ligands binding to a complex of two type I and two type II transmembrane receptors that regulate downstream target genes through phosphorylation of Smad proteins (Kondo, 2007; Little and Mullins, 2009). Two main interacting ligands of the pathway are Bmp2b and Bmp4, both of which have a higher binding affinity to type I Bmp receptors, such as Bmpr1bb, compared with type II receptors (Little and Mullins, 2009; Tajer et al., 2021). We have previously shown that Bmp signaling is required in the endoderm to regulate a fibroblast growth factor (Fgf) signaling response in the forming pouches (Lovely et al., 2016). Chemical inhibition of Bmp signaling impairs the morphogenesis of both pouches and the anterior pharyngeal endoderm as the area of endoderm anterior to the first pouch, resulting in craniofacial malformations (Lovely et al., 2016). These observations showed that functional Bmp signaling is indispensable for establishing proper endoderm morphology necessary for uninterrupted craniofacial skeleton patterning.

As a complex pathway that is essential for craniofacial morphogenesis, we hypothesized that Bmp signaling is potentially ethanol sensitive. Here, we tested the ethanol sensitivity mutations in several components of the Bmp pathway in zebrafish. We show here that hemi- or homozygous mutants for the Bmp ligand genes *bmp2b* and *bmp4* and for the receptor gene *bmpr1bb* (hereafter referred as Bmp mutants) predisposed zebrafish embryos to distinct ethanol-induced craniofacial shape changes, particularly jaw malformations. By using quantitative morphometrics, we show that ethanol-induced disruptions to anterior pharyngeal endoderm shape, which mirror changes in facial shapes, altered the expression domain of the oral ectoderm marker *fgf8a* as associated with jaw malformations. We go on to show, using fluorescent analyses, that Bmp signaling responses were lost specifically in the endoderm of Bmp mutants but that ethanol does not impact Bmp signaling in any meaningful way. Genetic analysis showed that − following ethanol exposure − *BMPR1B* associates with jaw deformations in children, which mirrors our zebrafish data and underlines the predictive strength of our zebrafish findings. Collectively, our data linked perturbations in Bmp signaling to ethanol susceptibility during craniofacial development in zebrafish and human, establishing mechanistic concepts in gene−ethanol interactions for future studies of FASD.

## RESULTS

### Mutations in multiple components of the Bmp pathway sensitize embryos to ethanol-induced viscerocranial malformations

To build on previous work, where we identified multiple ethanol-sensitive genetic loci that predisposed to ethanol-induced craniofacial malformations (McCarthy et al., 2013; Swartz et al., 2014, 2020), we performed a candidate screen to identify additional ethanol-sensitive genes. From this screen, we identified the Bmp-signaling pathway component genes − pathway ligands *bmp2b* and *bmp4* (Mullins et al., 1996; Stickney et al., 2007), and pathway receptor *bmpr1bb* (Neumann et al., 2011) − as being ethanol-sensitive genes that regulate facial development. Our previous work showed that Bmp signaling is required for facial development by regulating pharyngeal endoderm morphogenesis at 10-18 h post fertilization (hpf) (Lovely et al., 2016). We also showed that the Bmp ligand genes *bmp2b* and *bmp4* are expressed adjacent to the development endoderm during this time window (Lovely et al., 2016). In addition, both Bmp2b and Bmp4 have a higher binding affinity for type I Bmp receptors over type II Bmp receptors (Little and Mullins, 2009; Tajer et al., 2021), linking *bmpr1bb*. This, ultimately, provides a testable mechanism for the ethanol-induced craniofacial defects we examine below.

To test these Bmp mutants, we originally exposed zebrafish embryos to a sub-phenotypic dose of 1% ethanol (v/v) at time points between 6 hpf and 5 days post fertilization (dpf). We selected 1% ethanol (v/v) as the highest applicable dose not causing craniofacial defects in wild-type embryos, as higher doses of ≥1.25% ethanol had been shown to impact facial development (Bilotta et al., 2004; Everson et al., 2023; McCarthy et al., 2013; Swartz et al., 2014; Zhang et al., 2014). Within 5 min of exposure, a concentration of 1% ethanol (v/v) equilibrates to an average ethanol in-tissue concentration of 50 mM (∼30% of the medium) (Flentke et al., 2014; Lovely et al., 2014; Reimers et al., 2004; Zhang et al., 2013). This, importantly, is roughly equivalent to a blood alcohol concentration of 0.23% in humans; while a binge dose is physiologically relevant to FASD and humans are readily capable of surpassing this amount (Canfield et al., 2019; Ethen et al., 2009; Jones, 2008; Maier, 2001; Whaley et al., 2019).

**Fig. 1.**
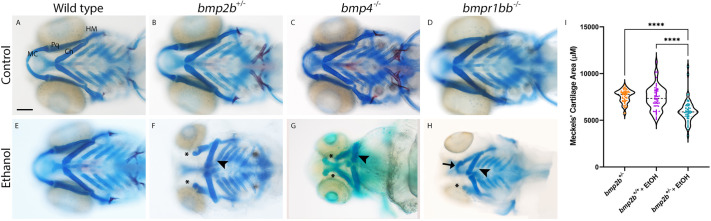
**Multiple members of the Bmp pathway display ethanol-sensitive facial phenotypes.** (A-H) Whole-mount images showing the viscerocranium of zebrafish larvae at 5 dpf that had been exposed to ethanol (Ethanol) or not (Control). Cartilage is shown in blue, bone in red. Views are ventral, with anterior to the left. Scale bar: 100 μm. MC, Meckel's cartilage; Pq, palatoquadrate cartilage; Ch, ceratohyal cartilage; HM, hyomandibular cartilage. *bmp2b^+/−^* or *bmp4^−/−^* or *bmpr1bb^−/−^* larvae develop comparable to wild-type larvae (A-D). Exposure to 1% ethanol at 10-18 hpf results in a range of defects to the viscerocranium, from loss of MC at the extreme end of this range (asterisks) to reductions in size and changes in shape in the MC (arrow) as well as a flattening of the Ch (arrowheads) (E-H). The average ethanol-induced defects are seen in Bmp mutant alleles but not their wild-type siblings. (I) Violin plot showing area measures of Meckel's cartilage. The size of Meckel's cartilage elements is reduced in ethanol-treated *bmp2b^+/−^* larvae compared to ethanol-treated wild-type or untreated *bmp2b^+/−^* larvae, with *F*=36.85, ****P=0.0001, one-way ANOVA, *n*=29 larvae, both Meckel's cartilage elements per group (*n*=58 in total).

By using this long exposure paradigm, we found that mutations in our chosen Bmp mutants sensitize developing zebrafish to a range of ethanol-induced developmental defects, including small eyes, defects to bone mineralization and, relevant for this study, jaw malformations (Fig. 1A-H). While *bmp2b*^−/−^ embryos do not develop past 16 hpf (Nguyen et al., 1998), heterozygous *bmp2b^+/−^*, *bmp4^−/−^* or *bmpr1bb^−/−^* larvae undergo normal craniofacial development and are − superficially − indistinguishable from their wild-type siblings (Fig. 1A-D). However, when exposed to ethanol under our conditions of 1% ethanol (v/v), these Bmp mutants developed a range of jaw defects, ranging from malformations to outright absence of the jaw (Fig. 1E,F). The expressivity of the spectrum of jaw phenotypes was consistent between the different mutant lines with only the penetrance changing between the lines (Fig. 1E,F, Table 1). Jaw malformations were more common than absent jaw in ethanol-treated Bmp mutants, with 15.9% vs 4.6% in *bmp2b^+/−^*, 37.8% vs 6.13% in *bmp4^−/−^* and 29.1% vs 2.7% in *bmpr1bb^−/−^* embryos, with variation between experimental groups (Table 1). Our quantifications also demonstrated that while wild-type siblings never displayed sensitivity to ethanol, heterozygous *bmp4^+/−^* and *bmpr1bb^+/−^* larvae were ethanol sensitive with incomplete penetrance (Table 1). These results revealed that consistent viscerocranial malformations − in particular jaw absence − occur in ethanol-treated zebrafish carrying Bmp pathway mutations, but not in Bmp-mutant fish that had not been exposed to ethanol or wild-type fish treated with ethanol.

**Table 1.** Penetrance of gene−ethanol interactions in Bmp mutants

Experiment	Untreated embryos	Jaw malformations	Penetrance [in %]	EtOH-treated embryos	Jaw malformations	Penetrance [in %]	Jaw loss	Penetrance [in %]
***bmp2b***								
1	37	0	0.0	41	6	14.6	0	0.0
2	45	0	0.0	41	10	24.4	3	7.3
3	37	0	0.0	115	20	17.4	4	3.5
4	80	0	0.0	46	9	19.6	5	10.9
5	64	0	0.0	61	12	19.7	1	1.6
6	75	0	0.0	193	26	13.5	7	3.6
7	23	0	0.0	24	0	0.0	4	16.7
Total	361	0	0.0	521	83	15.9	24	4.6
***bmp4***								
1	24	0	0.0	63	30	47.6	3	4.8
2	41	0	0.0	131	38	29.0	2	1.5
3	42	0	0.0	38	24	63.2	2	5.3
4	51	0	0.0	94	20	21.3	16	17.0
5	44	0	0.0	82	42	51.2	2	2.4
Total	202	0	0.0	408	154	37.7	25	6.1
***bmpr1bb***								
1	105	0	0.0	82	41	50.0	1	1.2
2	48	0	0.0	38	1	2.6	0	0.0
3	154	0	0.0	69	13	18.8	4	5.8
Total	307	0	0.0	189	55	29.1	5	2.6

Percentage of jaw malformations and jaw loss per experiment in ethanol-treated Bmp single-mutant embryos generated from random heterozygous crosses.

From this initial long exposure paradigm, we were able to narrow down the exposure window from 10-18 hpf−the same window when Bmp signaling is required for endoderm morphogenesis and jaw development (Lovely et al., 2016) – to exposure before 10 hpf, without adding to the penetrance and expressivity of the phenotypic spectrum in each single mutant (C.B.L., personal observation). We chose to work on *bmp4* larvae, i.e. *bmp4^+/−^* and *bmp4^−/−^* larvae, and their wild-type siblings, in all subsequent experiments as the mutation of *bmp2b* is weakly dominant (Kishimoto et al., 1997) and *bmpr1bb* is of the WIK (https://zfin.org/ZDB-GENO-010531-2) genetic background, whereas all other Bmp mutants used in this study are of the AB (https://zfin.org/ZDB-GENO-960809-7) genetic background, both of which were used in our additional analyses. In addition, we analyzed *bmp4* larvae, as they showed the greatest variation in response to ethanol treatment (Table 1). To test the onset of ethanol-induced facial malformations, we started the ethanol exposure paradigm on wild-type and *bmp4^−/−^* embryos at 10 hpf, 14 hpf and 18 hpf. Our analysis showed a decrease in the percentage of jaw loss and jaw malformation when ethanol exposure was started at later developmental stages (Table S1). When starting our ethanol exposure paradigm at 24 hpf, we observed no jaw loss and only 1.2% of embryos with jaw malformations (Table S2). To test if dosage was the determining factor for the lack of ethanol-induced craniofacial malformations at 24 hpf, we repeated the experiments starting our exposure paradigm at 24 hpf but increased our ethanol exposure concentration from 1% to 1.3%. We did not see any increase in the penetrance and expressivity of craniofacial malformations compared to the 1% exposure dose (Table S2). Combined, these results suggest that mutation in *bmp2b*, *bmp4* and *bmpr1bb* sensitizes embryos to ethanol-induced facial defects when exposed to ethanol at 10-18 hpf.

### Ethanol alters overall shape of the viscerocranium in Bmp mutants

Micrognathia is a hallmark of ethanol exposure in humans, although recent data have shown greater variation in facial shape in FASD (Blanck-Lubarsch et al., 2020; Suttie et al., 2013). To understand the impact of ethanol on viscerocranial shape and size, we undertook a series of quantitative measures to directly assess facial shape changes in zebrafish. To quantify micrognathia-like reductions in jaw size observed in FASD, we dissected and measured the size of the Meckel's cartilages from untreated *bmp2b^+/−^* and ethanol-treated wild-type and *bmp2b^+/−^* larvae. Ethanol-treated *bmp2b^+/−^* larvae displayed a significant reduction in jaw size compared to that of untreated *bmp2b^+/−^* and ethanol-treated wild-type larvae (Fig. 1I, *n*=29 larvae, both Meckel's cartilage elements per group (58 total), one-way ANOVA, *F* ratio (*F*)=36.85, *P*<0.0001). Beyond jaw defects, we observed malformations in additional viscerocranial cartilage elements, in particular an increase of the angle between the ceratohyal (Ch) elements (Fig. 1, compare panels B-D with F-H). This suggests that embryonic ethanol exposure is disrupting facial shape.

**Fig. 2.**
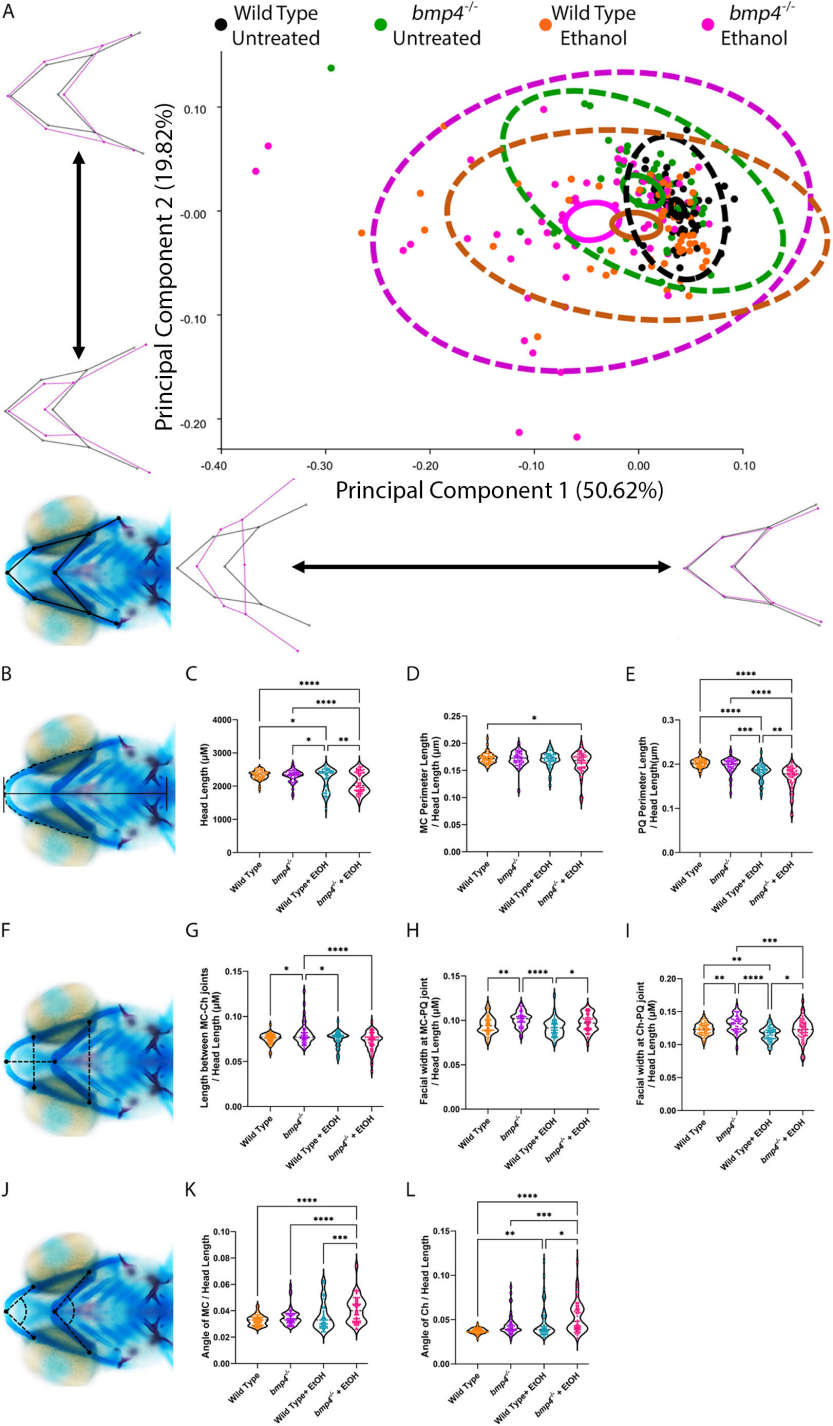
**Ethanol exposure alters viscerocranial shape in *bmp4^−/−^* zebrafish larvae.** (A,B,F) Whole-mount images of the viscerocranium in 5 dpf larvae showing landmarks, linear measures and cartilage angles. Cartilage is shown in blue, bone in red. Views are ventral, with anterior to the left. (A) Landmarks were placed on several joints between the cartilage elements of the viscerocranium. Genotypes are color-coded, with black indicating untreated wild-type larvae (*n*=61), green indicating untreated *bmp4*^−/−^ larvae (*n*=54), orange indicating ethanol-treated wild-type larvae (*n*=58) and magenta indicating ethanol-treated *bmp4*^−/−^ larvae (*n*=66). Areas surrounded by dashed lines represent 95% confidence ellipses comprising all individual data points for each group, areas surrounded by solid lines represent 95% confidence ellipses comprising the mean data points for each group. Wireframe graphs represent the variation as specified at each axis, with black representing no variation and magenta representing variation relative to the black wireframe. For example, principal component (PC)1 captures a shortening and widening in viscerocranial shape, while PC2 represents variation in midfacial width. Procrustes ANOVA showed significant a change in the viscerocranial shape (*F*=10.37, d.f.=36, *P*=0.0001). (C) Violin plot of overall head length. Subsequent measurements are all plotted as ratio to overall head length. (D) Meckel's cartilage (MC) perimeter to head length. (E) Palatoquadrate (PQ) cartilage perimeter to head length. (G) Length between midline MC-ceratohyal cartilage (Ch) joints to head length. (H) Facial width at MC-PQ cartilage joints to head length. (I) Facial width at Ch-PQ cartilage joints to head length. (K) MC angle joint to head length. (L) CH angle joint to head length. Linear measures show a significant decrease in facial width and length, and an increase in the angle between cartilage elements, which is represented as flattening of the facial skeleton even when head length is taken into account. *P<0.05; **P<0.01; ***P<0.001; ****P<0.0001 (for individual graph statistics see Table S3).

PAE is known to result in general growth retardation and developmental delay in both humans and in animal models (Everson and Eberhart, 2023; Popova et al., 2023). To expand our assessment of Bmp−ethanol interactions on facial shape and control for developmental delays, we performed morphometric analysis on untreated and ethanol-treated wild-type and *bmp4^−/−^* larvae. This approach took into account changes in size when analyzing facial shape by using by Procrustes superimposition, which removes variation in size, position and orientation, key to our analyses in overall facial shape (Goodall, 1991; Klingenberg, 2011). We determined facial shape by labeling each joint in the viscerocranium (Fig. 2A, see Alcian Blue staining). Principal component analysis (PCA) of facial shape revealed that principal component (PC)1 represents >50% of facial variation as a shortening and widening of the viscerocranium, as well as a flattening of the angles of several cartilage elements (Fig. 2A, see Principal Component 1). PC2 represented almost 20% of the variation in facial shape, affecting width of the midface (Fig. 2A, see Principal Component 2). Our dataset shows that the greatest amount of variation occurred in ethanol-treated *bmp4^−/−^* larvae, while the smallest variation occurred in wild type, i.e. wild-type larvae (Fig. 2A, magenta vs black 95% confidence ellipses, i.e. areas surrounded by dashed lines). Ethanol-treated wild-type and untreated *bmp4^−/−^* larvae displayed similar increases in variation compared to untreated wild-type larvae, but less than ethanol-treated *bmp4^−/−^* larvae. The mean of each group, displayed as a solid-line surrounded ellipse centered within the 95% confidence ellipse, shows little overlap between the groups (Fig. 2A). Procrustes ANOVA analysis showed that these shape changes were significant (*F*=10.37, *P*<0.0001). Combined, our morphometric data set shows that ethanol-treated *bmp4^−/−^* larvae displayed significant variation in facial shape; however, either ethanol or *bmp4^−/−^* alone also increased the variation in viscerocranial shape compared to untreated wild-type larvae. We did not identify this variation in ethanol or *bmp4^−/−^* alone in our initial visual screens. Overall, these data showed that, while both ethanol-treated wild-type and untreated *bmp4^−/−^* larvae display greater variation of facial shape than untreated wild-type larvae, ethanol-treated *bmp4^−/−^* larvae exhibit the greatest variation in facial shape, with significant quantifiable changes in facial size and shape compared to all other groups. This suggests that ethanol- or mutation-induced craniofacial shape changes cannot be readily identified in visual screens for gross morphology, and that Bmp mutation appear to potentiate ethanol-induced facial shape changes.

To confirm the morphometric data, we performed linear measurements on the viscerocrania of the *bmp4* morphometric dataset. For genotyping we removed the tail of the larvae, preventing overall body length measures. However, we measured the overall length of the head as an indicator for general developmental delays. We also measured the length between the midline joints of Meckel's and ceratohyal cartilages, the width between the joints of Meckel's and palatoquadrate cartilages, and between ceratohyal and palatoquadrate cartilages, and the perimeter of Meckel's and palatoquadrate cartilages (Fig. 2B,F). We further performed angle measurements of the midline joints of Meckel's and ceratohyal cartilages to analyze the flattening of the viscerocranium cartilage elements (Fig. 2J). We observed a significant decrease in head length due to ethanol, which was further exacerbated by loss of *bmp4* (Fig. 2C, Table S3). While we observed only a significant decrease in the perimeter of Meckel's cartilages between untreated wild-type and ethanol-treated *bmp4^−/−^* larvae, we observed significant decreases in the perimeter of palatoquadrate cartilages in ethanol-treated *bmp4^−/−^* larvae compared to all other groups (Fig. 2D,E, Table S3). Consistent with PC1 (Fig. 2A), we observed a significant decrease in length between Meckel's and ceratohyal cartilages in ethanol-treated *bmp4^−/−^* larvae compared to untreated *bmp4^−/−^* larvae (Fig. 2G, Table S3). We observed significant changes in the facial width at both Meckel's and palatoquadrate cartilages, and ceratohyal and palatoquadrate cartilages (Fig. 2H and I, respectively, Table S3). Untreated *bmp4^−/−^* larvae showed an increase in width for both measurements compared to that of untreated and ethanol-treated wild-type larvae (Fig. 2H,I, Table S3), while showing an increase in the width at ceratohyal and palatoquadrate cartilages compared to that of ethanol-treated *bmp4^−/−^* larvae (Fig. 2I, Table S3). Ethanol-treated *bmp4^−/−^* larvae showed significant increases in width at Meckel's and palatoquadrate cartilages compared to ethanol-treated wild-type larvae (Fig. 2H, Table S3), and increases in width at ceratohyal and palatoquadrate cartilages compared to both untreated and ethanol-treated and wild-type larvae (Fig. 2I, Table S3). Angle measurements of Meckel's and ceratohyal cartilages in ethanol-treated *bmp4^−/−^* larvae showed significant increases in cartilage angles (Fig. 2K,L, Table S3), consistent with the flattening of the viscerocranial cartilages observed in initial screens (Fig. 1), and PC1 and PC2 of our morphometric analysis (Fig. 2A). Overall, our morphometric measurements document that the Bmp−ethanol interaction results in a smaller jaw, and a shorter and wider viscerocranial shape, which is consistent with FASD in humans (Bemquerer et al., 2022; Suttie et al., 2013).

### Combinatorial loss of Bmp pathway components exacerbates ethanol-induced viscerocranial malformations

**Fig. 3.**
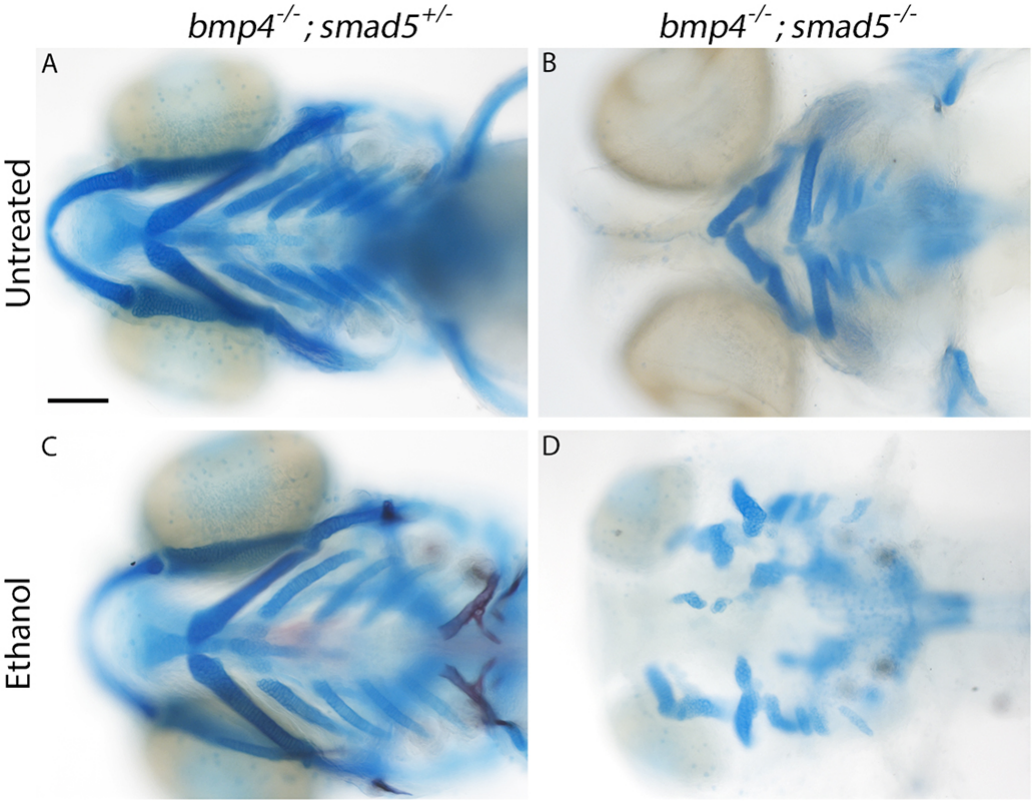
***bmp4^−/−^;smad5^−/−^* zebrafish larvae exhibit fully penetrant, exacerbated ethanol-induced facial malformations.** (A-D) Whole-mount images showing the viscerocranium of zebrafish larvae at 5 dpf that had been exposed to ethanol (Ethanol) or not (Untreated). Cartilage is shown in blue, bone in red. Views are ventral, with the anterior to the left. Scale bar: 100 μm. (A) No impact on facial formation was observed in untreated *bmp4^−/−^;smad5^+/−^* larvae (A). Stereotypical *smad5* mutant phenotypes were observed in untreated *bmp4^−/−^;smad5^−/−^* larvae (B). Ethanol does not impact viscerocranial morphology in *bmp4^−/−^;smad5^+/−^* larvae (C), but malformations in ethanol-exposed *bmp4^−/−^;smad5^−/−^* larvae (D) are fully penetrant and ethanol sensitive, resulting in several viscerocranial malformations, thereby recapitulating the severe phenotypes observed in dorsomorphin-treated embryos (Lovely et al., 2016).

While we consistently observed ethanol-induced viscerocranial shape changes in tested Bmp mutants that differed from wild-type controls, both penetrance and expressivity of these defects were highly variable (Figs 1 and 2, Table 1). The Bmp pathway is a complex signaling pathway comprising multiple components (Little and Mullins, 2009). These components can be partially redundant at different levels of the pathway (Li et al., 2011; Mu et al., 2021; Schille et al., 2016; Wise and Stock, 2010). Given this redundancy, we hypothesized that a combinatorial loss of pathway components increases both the penetrance and expressivity of ethanol-induced viscerocranial defects. Using a hypomorphic *smad5* allele that has a highly stereotyped phenotype of cartilage fusions and malformations and that has been previously shown to be insensitive to ethanol exposure (McCarthy et al., 2013; Swartz et al., 2011), we generated double-mutant *bmp4^−/−^;smad5^−/−^* larvae (Fig. 3). We found that *bmp4^−/−^;smad5^−/−^* larvae exhibit 100% penetrant ethanol-induced malformations that are i) more severe than the stereotypical *smad5* phenotype in untreated *bmp4^−/−^;smad5^−/−^* larvae, ii) much more severe than those in any of the Bmp single mutants (Fig. 3D compared to B; Figs 3B and Fig. 1H-F) and iii) highly reminiscent of the severe facial phenotypes observed in dorsomorphin-treated embryos (Lovely et al., 2016). We also observed a spectrum of phenotype severity in *bmp4^+/−^;smad5^−/−^* larvae, in which one copy of *bmp4* remained wild-type: these phenotypes range from stereotypical *smad5* mutant phenotypes to phenotypes comparable to ethanol-treated double-homozygous *bmp4^−/−^;smad5^−/−^* larvae (Fig. S1). In contrast, we observed no impact of ethanol on double-heterozygous *bmp4^+/−^;smad5^+/−^* larvae (C.B.L, personal observation). These genetic interaction results indicate that the variation in *bmp^+/−^;smad5^−/−^* larvae may be driven either by expression differences of other components of the Bmp pathway or by additional yet identified ethanol-sensitive alleles. Ultimately, these data support our previous observation that loss of *bmp4* was able to potentiate ethanol-induced facial shape changes, with the most significant impact observed when mutation and ethanol exposure were combined.

### Ethanol-treated Bmp mutants display malformation of the anterior pharyngeal endoderm and *fgf8a* expression in the oral ectoderm

**Fig. 4.**
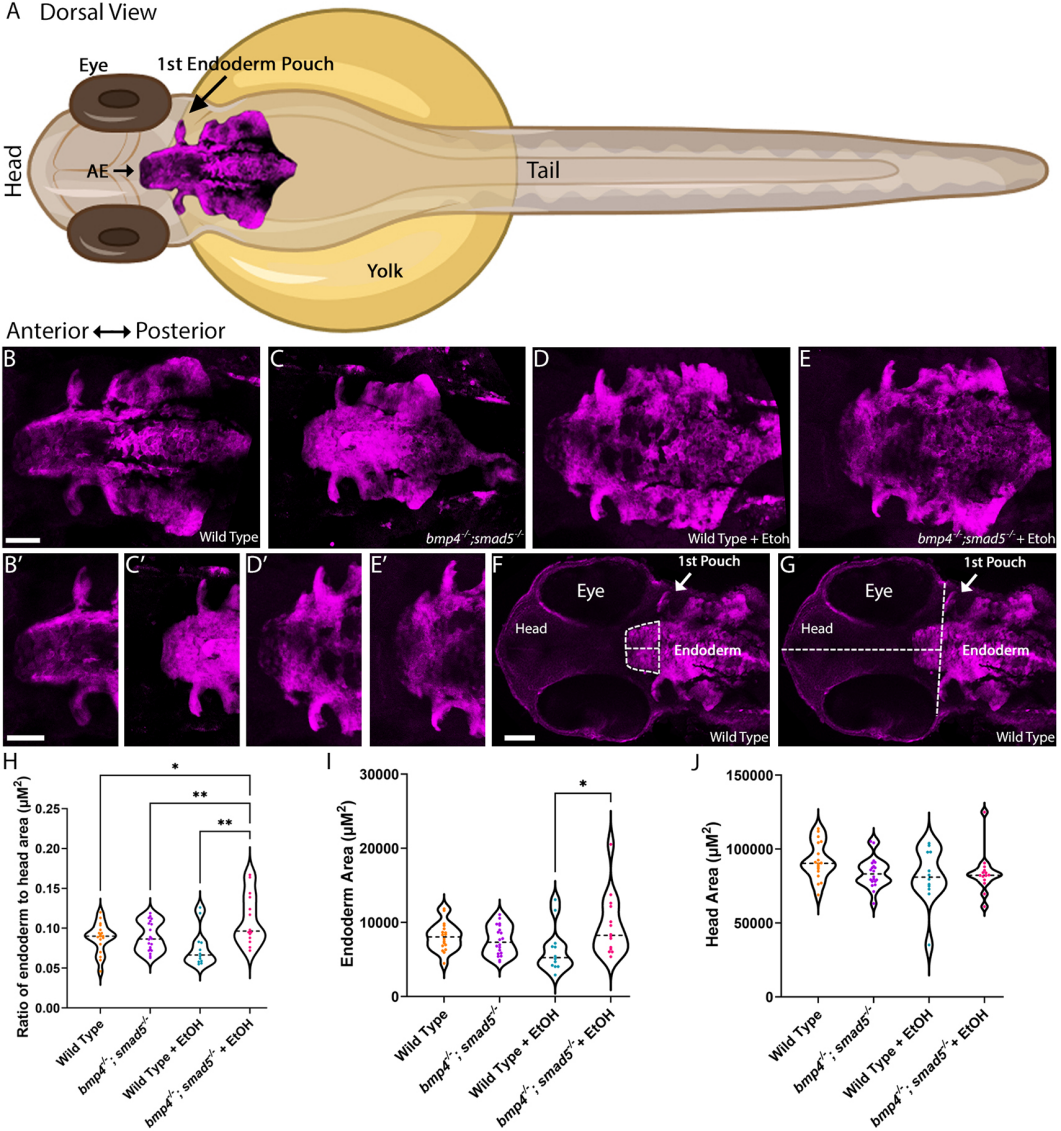
**Ethanol exposure alters area of the anterior pharyngeal endoderm in *bmp4^−/−^;smad5^−/−^* zebrafish embryos.** (A) Schematic of a zebrafish embryo with annotation of the pharyngeal endoderm. AE, anterior endoderm. The zebrafish image was generated using BioRender Created in BioRender by Lovely, B., 2025. https://BioRender.com/20hca0u. This figure was sublicensed under CC-BY 4.0 terms. (B-E) Whole-mount images of the pharyngeal endoderm in zebrafish embryos at 36 hpf. (B′-E′) Magnified views of the anterior pharyngeal endoderm from panels B-E, respectively. Imaged were untreated wild-type embryos, *n*=18 (B,B′); untreated *bmp4^−/−^;smad5^−/−^* embryos, *n*=20 (C,C′); ethanol-treated wild-type embryos, *n*=12 (D,D′); and ethanol-treated *bmp4^−/−^;smad5^−/−^* embryos, *n*=14 (E,E′). Views are dorsal, with the anterior to the left. (F,G) Whole-mount images of untreated wild-type zebrafish heads taken at 36 hpf, showing the area and length of the anterior pharyngeal endoderm measured from the first pouch to the anterior-most tip. The width of the anterior pharyngeal endoderm was measured at the level of the first pouch (F). The head area was calculated from the length of the head (first pouch to most-anterior tip of the head) and the width of the head (measured at level of the first pouch) (G). All scale bars: 50 μm. (H) Violin plots showing the ratio of the anterior pharyngeal endoderm to the head area (H), the endoderm area (I) or the head area (J). Ethanol-treated *bmp4^−/−^;smad5^−/−^* embryos show increased area of the anterior endoderm compared to all other groups (H). This increased size is not due to changes in head size (I) but directly due to increased size of the anterior pharyngeal endoderm (J). Individual graph statistics are provided in Table S4.

We have previously shown that zebrafish embryos treated with the Bmp signaling inhibitor dorsomorphin at 10-18 hpf exhibit disrupted morphogenesis of the anterior pharyngeal endoderm (Lovely et al., 2016). Multiple studies have shown that disruptions to the anterior pharyngeal endoderm leads to jaw malformations (Balczerski et al., 2012; Couly et al., 2002; Crump et al., 2004; Haworth et al., 2004, 2007). To analyze anterior pharyngeal endoderm shape, we labeled the pharyngeal endoderm by crossing the *sox17:EGFP* transgene that labels all endoderm with EGFP (Chung and Stainier, 2008), into the *bmp4^−/−^;smad5^−/−^* background. Compared to wild type, ethanol-treated *bmp4^−/−^;smad5^−/−^* embryos showed mild changes to overall endoderm shape (Fig. 4B-E). However, our more detailed examination showed that the shape of the anterior pharyngeal endoderm is altered in ethanol-treated *bmp4^−/−^;smad5^−/−^* embryos (Fig. 4B′-E′). To quantify these changes in anterior pharyngeal endoderm shape, we measured the width at the first pouch (AE width), the midline length (AE length) and area anterior pharyngeal endoderm (AE area) from the first pouch to the anterior-most end of the pharyngeal endoderm (Fig. 4F). We normalized for ethanol-induced general growth deficits of the anterior pharyngeal endoderm shape, by measuring the length and width of the embryonic head and calculating head area (Fig. 4G). Calculating head area instead of directly measuring head area controls for size changes due to ethanol-induced reductions in eye size and did not show any difference between groups (Fig. 4H). Our analyses showed a significant increase in AE area expressed as a ratio to head area in ethanol-treated double-homozygous *bmp4^−/−^;smad5^−/−^* embryos compared to all other groups (Fig. 4H, Table S4). AE area alone of ethanol-treated *bmp4^−/−^;smad5^−/−^* embryos only exhibited a significant increase compared to ethanol-treated wild type, wild-type embryos (Fig. 4I, Table S4). We also observed non-significant increases in AE length and AE width, expressed as a ratio to head area, in ethanol-treated *bmp4^−/−^;smad5^−/−^* embryos, while no differences were observed in head length and width (Fig. S2, Table S5). Collectively, our observations in zebrafish document that changes of the anterior pharyngeal endoderm area in ethanol-treated Bmp mutants might underlie viscerocranial malformations.

**Fig. 5.**
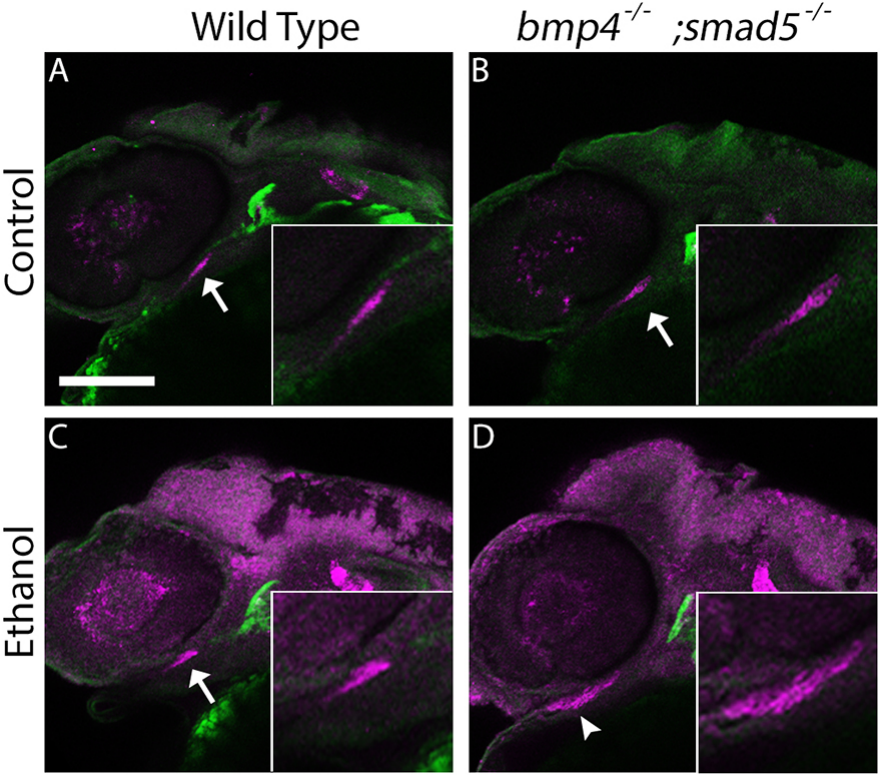
**Ethanol exposure changes shape of oral ectoderm expression domain in *bmp4^−/−^;smad5^−/−^* zebrafish embryos.** (A-D) Whole-mount confocal images of untreated (top panels) or ethanol-treated (bottom panels) wild-type (left) or *bmp4;smad5;sox17:EGFP* (right) zebrafish embryos fluorescence labeled for *fgf8a* (magenta) gene expression at 36 hpf. Views are lateral, with anterior to the left. Scale bar: 100 μm. The endoderm is labeled with GFP (green). Arrows in A-C indicate normal expression of *fgf8a* in the oral ectoderm of untreated wild-type and *bmp4^−/−^;smad5^−/−^* embryo as well as ethanol-treated wild-type embryos. Arrowhead in D indicates that the domain of *fgf8a* expression in ethanol-treated *bmp4^−/−^;smad5^−/−^* embryos is expanded anteriorly (*n*=7 embryos per group). Panel insets are magnified (10×) views of the indicated *fgf8a* expression domains.

Previous work has shown that the anterior pharyngeal endoderm is necessary to induce signaling factor expression in the oral ectoderm (Balczerski et al., 2012; Haworth et al., 2004, 2007). This suggests that blocking Bmp signaling with either dorsomorphin or ethanol will disrupt expression of the critical oral ectoderm markers, such as *fgf8a* and *pdgfaa.* To test if expression of *fgf8a* or *pdgfaa* is altered in dorsomorphin-treated zebrafish embryos, we performed HCR-based, fluorescent *in situ* hybridization. In wild-type embryos, *fgf8a* is expressed in a small domain in the oral ectoderm, ventro-posterior to the developing eye and retina (Fig. S3A). Wild-type embryos treated with dorsomorphin at 10-18 hpf, i.e. the same developmental time window as our ethanol-exposure paradigm, have variable defects in *fgf8a* expression, from loss of expression to anterior expansion of expression to the level of developing the retina (Fig. S3B,C). This mirrors changes in the expression levels of *fgf8a* in other anterior pharyngeal endoderm mutants (Balczerski et al., 2012; Haworth et al., 2007). Interestingly, expression of *pdgfaa*, which is expressed throughout the oral ectoderm, is unaltered in dorsomorphin-treated embryos (Fig. S3D,E), demonstrating that the oral ectoderm is not lost when Bmp signaling is attenuated and that the endoderm mediates the expression of some, but not all, oral ectodermal signaling molecules. We then tested if expression of *fgf8a* is altered in ethanol-treated *bmp4^−/−^* and *bmp4^−/−^;smad5^−/−^* embryos. The expression domain of *fgf8a* was similarly well detectable in untreated *bmp4^−/−^*, *bmp4^−/−^;smad5^−/−^* and ethanol-treated wild-type embryos, with embryos from each genotype showing little difference to untreated wild-type embryos (Fig. S4A-C; Fig. 5A-C). Expression of *fgf8a* in ethanol-treated *bmp4^−/−^* embryos were subtly expanded anteriorly to the level of developing the retina, while *fgf8a* expression in *bmp4^−/−^;smad5^−/−^* embryos was markedly expanded anteriorly to the level of developing the retina (Fig. S4D; Fig. 5D). This anterior expansion of the *fgf8a* expression domain is similar to the anterior expansion of *fgf8a* we observed in dorsomorphin-treated embryos (Fig. S4D; Fig. 5D compared to Fig. S3C). Our data of ethanol-induced changes to the *fgf8a* expression domain in the oral ectoderm upon perturbation of Bmp signaling are consistent with previous work showing that malformations of the anterior pharyngeal endoderm disrupt oral ectoderm expression domains and subsequent viscerocranial malformations (Balczerski et al., 2012). While limited to expression of *fgf8* as a critical marker, these observations indicate that synergistic Bmp−ethanol perturbations disrupt an anterior pharyngeal endoderm−oral ectoderm-signaling axis.

**Fig. 6.**
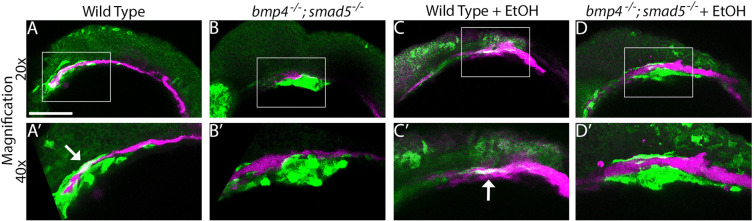
**Endoderm-specific Bmp signaling responses are lost in *bmp4^−/−^;smad5^−/−^* embryos but not in ethanol-treated zebrafish embryos.** (A-D′) Whole-mount, confocal images of *bmp4;smad5;sox17:EGFP;BRE:mKO2* wild-type or *bmp4^−/−^;smad5^−/−^* embryos at 36 hpf that had been ethanol treated (+EtOH) (C-D′) or not (A-B′). Ectoderms were fluorescence labeled for GFP (false-colored magenta) and for active Bmp signaling with mKO2 (false-colored green). Views are lateral, with anterior to the left. Boxed areas within A-D are shown magnified (40×) in A′-D′, respectively. Scale bar: 100 μm. Arrows in A′ and C′ indicate overlap of Bmp signaling response and *sox17*-labeled endoderm in wild-type embryos. Compared with wild-type embryos (A,A′,C,C′), the endoderm-specific Bmp signaling response is lost in *bmp4^−/−^;smad5^−/−^* embryos (B,B′,D,D′). Ethanol exposure does not alter the Bmp response (C-D′). Embryos per group: wild type (*n*=5); *bmp4^−/−^;smad5^−/−^* (*n*=4); wild type + EtOH, *n*=4; *bmp4^−/−^;smad5^−/−^* (*n*=8).

Our above data suggest that ethanol can reduce Bmp signaling responses in the anterior endoderm. To test this, we generated the *bmp4;smad5;sox17GFP;BRE:mKO2* double-mutant, double transgenic line that labels the endoderm with GFP and active Bmp signaling with monomeric Kusabira-Orange 2 (mKO2) (Collery and Link, 2011). We have previously used this BMP response element (*BRE):mKO2* line to analyze active Bmp responses during early development of the pharyngeal arches (Sheehan-Rooney et al., 2013). By using this line, we were able to show that Bmp signaling is lost in the pharyngeal endoderm of *bmp4^−/−^;smad5^−/−^* embryos, independently of ethanol exposure at 18 hpf, the end of Bmp signaling responses in the endoderm (Lovely et al., 2016) (Fig. 6B,B′ and D,D′). However, we did not observe any decreases in the BRE response in other pharyngeal tissues in response to ethanol exposure (Fig. 6A-D). We quantified BRE fluorescence levels by measuring the corrected total fluorescence of the BRE response within the pharyngeal arches and observed no significant changes in the BRE response in untreated *bmp4^−/−^;smad5^−/−^* embryos or ethanol-exposed wild-type embryos (Fig. S5, Table S6). This suggests that ethanol does not impact Bmp signaling but affects other targets in endoderm morphogenesis.

### Bmp−ethanol interactions translate to human FASD jaw volume

**Fig. 7.**
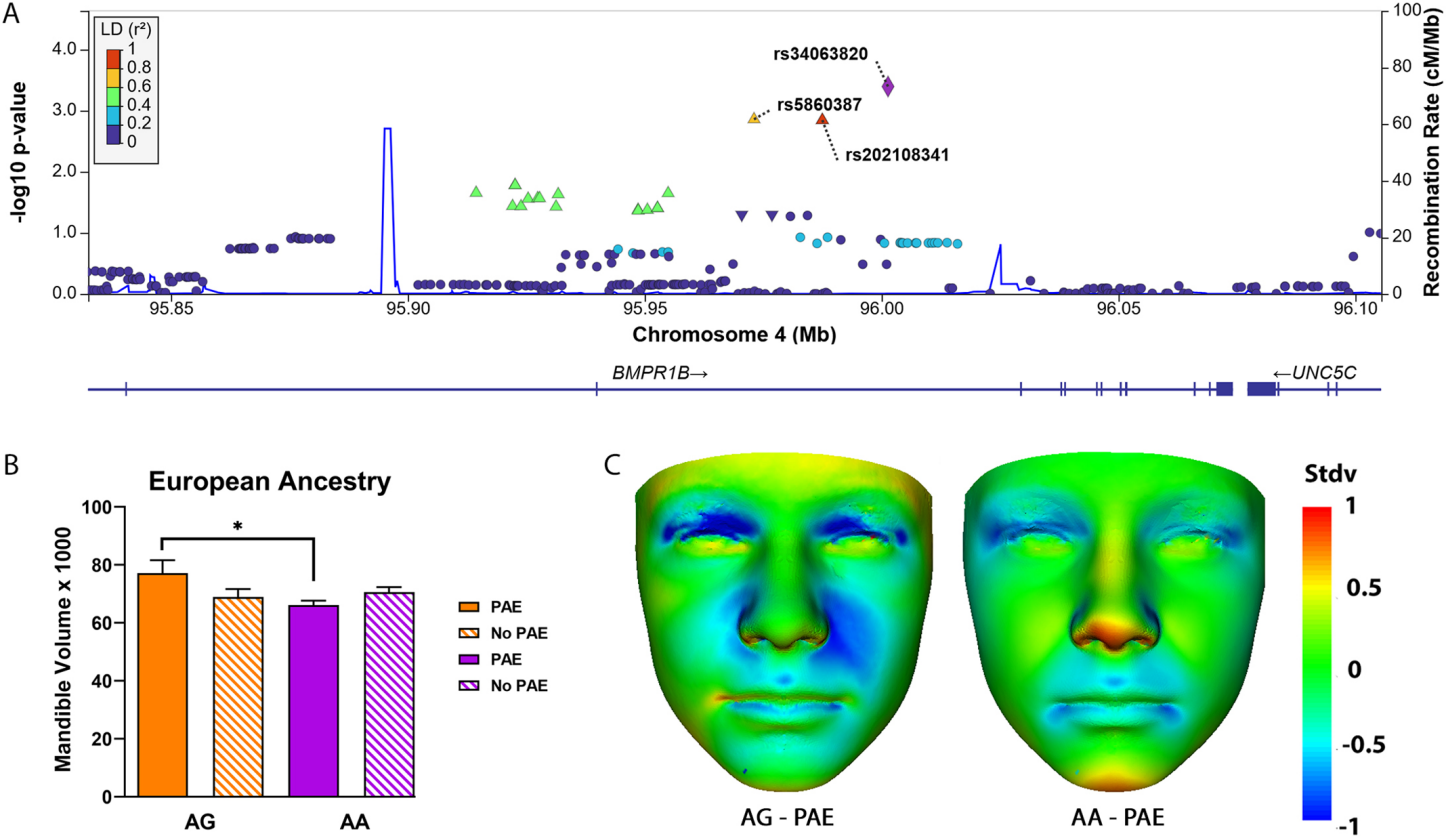
**Association of jaw deformations with single nucleotide polymorphisms (SNPs) located within *BMPR1B* in the European ‘look-up’ resource.** (A) The *y*-axis denotes the −log10 (*P*-value) for the genotype×PAE effect for association with mandible volume (triangles). Indicated on the *x*-axis is the physical position on the chromosome (Mb). The extent of linkage disequilibrium (LD) (as measured by *r^2^*) in the 1000 Genomes European reference panel between each SNP (triangles) and the purple diamond SNP is indicated by the color scale at the top left. Larger values of *r^2^* indicate a greater LD. rs34063820, the SNP with the highest *P*-value (*P*=4×10^−4^) in *BMPR1B*, is indicated by a purple diamond. Two SNPs in LD with *rs34063820* are also associated with mandible volume, i.e. *rs202108341*, *P*=8×10^−3^ (red triangle) and *rs5860387*, *P*=8×10^−3^ (yellow triangle). (B) Mandible volume in children of European ancestry (EA) with or without prenatal alcohol exposure (PAE and No PAE, respectively) according to genotype. In individuals with *AA* genotype, the mandible volume was significantly decreased (*P=0.032) in PAE vs No PAE individuals with the *AG* genotype. (C) Dense surface model (DSM) analysis of facial signature heatmaps, indicating surface to normal displacement at ±1 standard deviation (Stdv) for mean No PAE age-matched individuals of EA, where red-blue-green coloring indicates a reduction-expansion or agreement compared to the normalized group. Left: *AG* genotype with PAE (*n*=10). Right: *AA* genotype with PAE (*n*=83), both normalized against all EA age-matched individuals (*AG+AA* genotype) without PAE (*n*=35). Red coloring on the mandible tip indicates a mandibular retraction or micrognathia.

Zebrafish has been used successfully to model human developmental disorders (Lieschke and Currie, 2007; Santoriello and Zon, 2012) and we have previously shown that our zebrafish screens can predict gene−ethanol interactions in humans (McCarthy et al., 2013). We, therefore, sought to validate if our zebrafish model of Bmp−ethanol interactions impacting facial shape may be predictive of gene−ethanol associations in humans. From a study of children with and without PAE, we ran a genome-wide association study to explore if genotype was associated with anomalous mandible volume in the presence or absence of PAE (genotype×PAE interaction). This sample and the genotype×PAE results are utilized only as a ‘look-up’ resource – i.e. our small-sample size (total *n*=324) GWAS results are only used to look up human gene–ethanol interactions based on results from animal models – and not as a discovery sample (Dou et al., 2018; Hwang et al., 2024; McCarthy et al., 2013). The genome inflation factor for the genotype×PAE interaction was λ=1.049, indicating no large inflation of *P*-values. The quantile−quantile (QQ) plot is provided as Fig. S6. We examined results for common variants in human *BMP2*, *BMP4* and *BMPR1B* in individuals of European ancestry (EA) (*n*=184) and African ancestry (AA) (*n*=135), who also had data for mandible volume. Of the EA sample, one single nucleotide polymorphism (SNP) in the 5′ region of *BMP2* (*rs235710*) was not significant (*P*=0.029) after adjusting for five independent tests (corrected α=0.01). This SNP was not in linkage disequilibrium (LD) with any other SNPs in or near *BMP2* (D′=0; all *P*>0.74). Similarly, one SNP in the 5′ region of *BMP4* (*rs72680543*, *P*=0.043) did not meet criteria for significance (corrected value for six independent tests was α=0.0083). Furthermore, *rs72680543* was in high LD (D′=1.0) with all other SNPs (all *P*>0.19). The best *P*-value for SNPs in the BMP receptor gene *BMPR1B* was for *rs34063820* (*P*=4.0×10^−4^, corrected value for 103 independent tests was α=4.8×10^−4^; minor allele frequency (MAF) A>G allele=0.12; see Fig. 7A, purple diamond). The main effect of the *rs34063820* genotype on mandible volume was not significant (*P*=0.17). There was no evidence of a genotype×PAE association with the SNP in the AA sample (*P*=0.16; MAF=0.039). These MAFs are slightly higher than those reported in the database of Genotypes and Phenotypes (dbGaP) (EA MAF=0.0453 and AA MAF=0.0095). Due to the low power in the AA sample, only EA results are reported hereafter.

There were two SNPs in modest to high LD with *rs34063820*, demonstrating modest association with mandible volume in the EA sample [*rs202108341* (red triangle): *P*=8×10^−3^; *rs5860387* (yellow triangle): *P*=8×10^−3^; Fig. 7A]. Among children of EA without PAE, a Wilcoxon test revealed no difference in mandible volume between those with the *AG* vs *AA* genotype (*P*=1.0; all comparisons are shown in Fig. 7B). However, among EA individuals with PAE, those with *AA* genotype had smaller mandible volume than those with *AG* genotype (EA *P*=0.032; Fig. 7B). A facial signature heatmap of the mean alcohol-exposed *AA* genotype group and the mean alcohol-exposed *AG* genotype group – both normalized against 35 age-matched unexposed individuals of EA − visualizes this volumetric reduction in response to PAE, which is localized to the most anterior part of the mandible (Fig. 7C). This suggests that gene by ethanol interaction in the Bmp pathway, specifically in *BMPR1B*, potentially underlies the abnormal jaw size in FASD individuals, highlighting the predictive value of our mechanistic studies underlying FASD etiology in zebrafish. However, due to the small sample size, these results could be false positives, and need to be replicated with a larger, independent sample.

## DISCUSSION

Formation of the facial skeleton is driven by a complex, highly coordinated, three-dimensional process involving multiple tissues in the developing head (Knight and Schilling, 2006; Medeiros and Crump, 2012; Murillo-Rincón and Kaucka, 2020). Proper regulation of the cell movements and tissue dynamics within a single tissue and between different tissues are crucial for these events (Knight and Schilling, 2006; Medeiros and Crump, 2012; Murillo-Rincón and Kaucka, 2020). These cell behaviors are regulated by numerous signaling pathways and can be attenuated either genetically and/or environmentally, leading to a cascading effect that disrupts craniofacial development (Knight and Schilling, 2006; Medeiros and Crump, 2012; Murillo-Rincón and Kaucka, 2020). Prenatal ethanol exposure, leading to FASD, results in highly variable sets of facial skeleton phenotypes, including the jaw (Blanck-Lubarsch et al., 2020). Genetic risk factors are main drivers of FASD symptomology, providing insight into the cellular and molecular processes potentially disrupted in FASD (Lovely, 2020).

### Bmp−ethanol interactions

Here, we show that mutation of certain Bmp pathway components sensitize zebrafish embryos to ethanol-induced viscerocranial malformations. We found that, when exposed to ethanol at 10-18 hpf, mutations in *bmp2b*, *bmp4* or *bmpr1bb*, sensitized embryos to a range of ethanol-induced defects of Meckel's cartilages with both – later developmental initiation points of ethanol exposure and high doses of ethanol at the later developmental initiation points – resulting in fewer ethanol-induced facial defects. These defects covered a wide range, i.e. outright loss of cartilage elements to reductions in cartilage size, and were consistent between all three Bmp mutant lines. Using a morphometric approach, we observed broad changes to the facial shape in ethanol-treated Bmp mutants. Ethanol-treated larvae displayed a shorter and wider face relative to untreated controls, and a flattening of several cartilage element angles. Many of these variations had not been identified in our initial screens, supporting evidence that our current approach will identify subtle phenotypes impacting facial shape that would be missed when simply using linear measures of cartilage size (McCarthy et al., 2013; Swartz et al., 2014, 2020). However, the variation of phenotypes observed needs much further investigation to address the wide array of facial defects seen. Although not examined, heterozygous *bmp4* and *bmpr1bb* larvae will be critical in shedding light on the complex relationship between ethanol exposure and genotype. This, combined with expanded timing and dosage analyses, will provide further insight into the broader phenotypic mechanisms that occur in these ethanol-sensitive alleles. Equally important, these broad ethanol-induced facial shape changes in zebrafish are consistent with analyses of human faces, in which alcohol-exposed individuals showed broad and varied changes of the facial shape compared to age-matched controls (Bemquerer et al., 2022; Suttie et al., 2013). Overall, our data document that our morphometric analysis in zebrafish improve the rigor of identifying and quantifying ethanol-induced subtle facial shape changes, modeling the highly variable ethanol-induced changes to the facial shape established in human studies.

Our results suggest that ethanol increased craniofacial variation in wild-type zebrafish larvae and that loss of *bmp4* potentiated this interaction. Exacerbation of facial phenotypes in *bmp4^−/−^;smad5^−/−^* larvae demonstrated that dose-dependent reduction in Bmp–signaling drives ethanol-induced phenotypes. Surprisingly, ethanol exposure did not impact Bmp-signaling responses, albeit we observed subtle non-significant increases in Bmp-signaling responses at 18 hpf. This result was unexpected based on our facial and endoderm analyses, as we expected Bmp signaling to be reduced upon ethanol exposure. It is possible that we need to examine earlier time windows, as 18 hpf might be too late to observe ethanol-induced changes to Bmp signaling. It is also possible that the loss of Bmp-signaling responses within the endoderm masks any impact of ethanol on Bmp signaling. A counter-hypothesis is that ethanol does not impact Bmp signaling in any meaningful way during pharyngeal development and that ethanol acts on additional targets that regulate some aspects of endoderm morphogenesis and/or craniofacial development. In this case, ethanol would act either downstream of Bmp signaling, impinging on target gene expression/function or on a parallel pathway, independent of, and concomitant with, Bmp signaling.

Ethanol can impact a number of epigenetic mechanisms, including chromatin modifications (Zakhari, 2013). Recent work has shown that the protein arginine methyltransferase 1 (PRMT1) can mediate Bmp-dependent Smad phosphorylation and DNA methylation, thereby regulating bone formation and suture closure in mice; moreover, ethanol has been shown to regulate both PRMT1 protein expression and function (Hashimoto et al., 2017; Ye et al., 2023; Zhao et al., 2019). This suggests that these epigenetic changes can disrupt the expression of Bmp target genes directly by modulating pathway function and target gene expression, or indirectly through methylation patterns that target expression of Bmp–signaling pathway components and/or downstream targets. Additionally, these gene expression changes could extend beyond the Bmp pathway and its downstream targets, affecting parallel pathways necessary for craniofacial development. Ultimately, a comprehensive approach that combines transcriptomic, proteomic and metabolomic methodologies investigating downstream or parallel pathway targets will be needed to further elucidate the observed variations in Bmp−ethanol interactions during jaw formation.

### Impact of ethanol on pharyngeal endoderm morphogenesis

Work using multiple model systems has shown that disruption to pharyngeal endoderm morphogenesis leads to jaw defects (Balczerski et al., 2012; Couly et al., 2002; Crump et al., 2004; Haworth et al., 2004, 2007). This disruption alters the endodermal signaling centers that directly pattern the jaw, leading to jaw hypoplasia (Couly et al., 2002; Suzuki et al., 1999; Vieux-Rochas et al., 2010). These signaling centers are critical for neural crest cell survival, with disruptions of endoderm morphogenesis leading to the death of neural crest cells (Edlund et al., 2014; Johnson et al., 2011; Kopinke et al., 2006). We have previously shown that Bmp signaling is required for pharyngeal endoderm morphogenesis at 10-18 hpf − a time frame identical to our ethanol-sensitive time window − and that blocking Bmp signaling with dorsomorphin during this time window results in a wide range of defects to the viscerocranium (Lovely et al., 2016). In addition, we observed increased cell death in tissues adjacent to the pharyngeal endoderm where neural crest cells condense into the pharyngeal arches, suggesting that the Bmp-induced defects in endoderm morphogenesis are increasing cell death in the neural crest (Lovely et al., 2016). In our current study, we show that our Bmp−ethanol interactions alter the anterior pharyngeal endoderm shape, thereby increasing the area of the anterior pharyngeal endoderm relative to head size. Strikingly, this increase in anterior pharyngeal endoderm size stands at odds with the decreases we observed in jaw shape and size. This has been previously observed by using morpholino knockdown of *vgll2a*, which results in shorter and wider endodermal pouches but viscerocranial hypoplasia as a result of increased neural crest cell death (Johnson et al., 2011). It is possible that ethanol-induced increases in anterior endoderm size disrupt endodermal signaling centers, leading to neural crest cell death. This, in turn, decreases contribution of neural crest cells to the forming jaw, thereby decreasing its size. However, future work examining endoderm-induced death of neural crest cells needs to be properly controlled, as ethanol exposure is known to directly induce the death of neural crest cell (Dunty et al., 2001; McCarthy et al., 2013).

While the anterior pharyngeal endoderm signals directly to the cranial neural crest for cell survival, this signaling can also be indirect by inducing the expression of oral ectoderm signaling factors – a step that is critical for jaw formation (Balczerski et al., 2012; Haworth et al., 2004, 2007). These tripartite tissue interactions can be highly variable, with endoderm mutants showing both reduction and expansion in oral ectoderm signaling centers, and resultant hyper- and hypoplasia of the viscerocranium (Balczerski et al., 2012). Here, we show that, in addition to endodermal defects, blocking Bmp signaling with dorsomorphin led to variable disruption of *fgf8a* expression in the oral ectoderm. Strikingly, expression of *fgf8a* in the oral ectoderm was increased in ethanol-treated *bmp4^−/−^;smad5^−/−^* embryos. Although consistent with the increased size of the anterior pharyngeal endoderm, this stands at odds with the disruption of Bmp signaling, suggesting that Bmp−ethanol interaction disrupts jaw formation through an endoderm−oral-ectoderm−neural-crest-signaling axis. However, how this increase in both anterior pharyngeal endoderm and oral ectoderm signaling centers differs from dorsomorphin-induced inhibition of Bmp signaling and still results in a smaller jaw in ethanol-treated Bmp mutants remains unclear. Both, the oral ectoderm and its surrounding neural crest express a number of signaling factors that drive jaw and palate formation (Swartz et al., 2011). Changes in the expression domain of any of these factors could disrupt local interaction points between the neural crest and the oral ectoderm, thereby resulting in jaw malformations (Balczerski et al., 2012). Of great interest is the effect of these early developmental disruptions on subsequent cartilage outgrowth, raising several questions regarding cellular events of jaw outgrowth downstream these tripartite interactions. Our data support the hypothesis that Bmp−ethanol interactions disrupt a signaling axis between endoderm, oral-ectoderm and neural crest. However, more work is required to analyze the impact of ethanol on cell behaviors establishing these tripartite interactions, and how these interactions drive the cellular mechanisms in the oral ectoderm and neural crest during subsequent jaw formation.

### Zebrafish model gene−ethanol interactions in human FASD

We have previously shown that our zebrafish screens can predict gene−ethanol interactions in humans suggesting that our Bmp−ethanol interactions impacting facial shape may also be predictive of gene−ethanol associations in humans (McCarthy et al., 2013). Consistent with this − in the absence of ethanol − disruption of *Bmp2*/*BMP2* in mice/humans, respectively, result in a hypoplastic jaw (Chen et al., 2019; Sahoo et al., 2011). From a study of children recruited with and without PAE, we show here that SNPs in *BMPR1B* were significantly associated with ethanol-associated jaw malformations. Rather than the significance *P*-value threshold of 5×10^−8^ that is typically utilized in a Discovery genome-wide association study (GWAS), we corrected for the number of independent regions within each gene, since these genes were explicitly identified *a priori* and the results are only meant to be used as a translational resource to collaborators. We reported *P*-values from the additive genotype×PAE interaction rather than the false discovery rate (FDR) for similar reasons. As the human GWAS is based on a small sample, and significant results were only observed in the EA sample, the study needs to be replicated with a larger, independent sample.

Jaw hypoplasia is commonly observed in FASD (Basart et al., 2018; Blanck-Lubarsch et al., 2020; Suttie et al., 2013) but studying FASD in humans is incredibly challenging due to the complex interplay between genetic background and ethanol timing and dosage. Our results show that zebrafish analyses can model gene−ethanol associations in humans, strongly phenocopying both the malformation and the variation inherent in human data (McCarthy et al., 2013; Suttie et al., 2013, 2017). However, due to the paucity of human genetic studies of FASD, the use of sample sizes with sufficient power to detect association of multiple genes in one epistatic model is currently impossible. Thus, the zebrafish model remains a powerful, efficient method to simultaneously examine the effect of multiple genes on facial measures and to generate a deeper mechanistic understanding of these gene−ethanol interactions on craniofacial development. While future functional human and zebrafish analyses will need to test for the causal relationship and mechanistic underpinnings of ethanol-induced jaw deformations, our work strongly suggests that jaw malformations in FASD are, in part, due to disruptions to epithelial dynamics and signaling events.

Combined, our results show that zebrafish can predict Bmp−ethanol associations in human and provide a valuable model system for determining the ethanol-sensitive tissue events that contribute to facial defects in FASD. However, despite our increased understanding of the clinical impact of prenatal ethanol exposure, much remains to be learned of the cellular mechanisms underlying FASD (Lovely, 2020). Our work here provides some of the first evidence of gene−ethanol interactions altering epithelial dynamics in the complex, endoderm−oral ectoderm−neural crest-signaling axis leading to facial malformations. This expands our current understanding of ethanol-sensitive tissue dynamics in FASD and provides a conceptual framework for future FASD studies. Ultimately, our work generates a mechanistic paradigm of ethanol-induced structural birth defects and connects ethanol exposure with concrete cellular events that could be sensitive beyond the jaw.

## MATERIALS AND METHODS

### Zebrafish (*Danio rerio*) care and use

All zebrafish were raised and cared for using established IACUC protocols approved by the University of Louisville (Westerfield, 2007). Adult fish were maintained at 28.5°C with a 14/10-h light/dark cycle. The *bmp2b^tc300a^* (Mullins et al., 1996), *bmp4st^72^* (Stickney et al., 2007), *bmpr1bb^sw40^* (Neumann et al., 2011) and *smad5^b1100^* (Swartz et al., 2011), *sox17:EGFP^s870^* (Chung and Stainier, 2008) and *BRE:mKO2^mw40^* (Collery and Link, 2011) zebrafish lines have been previously described. Sex as a biological variable is not applicable at our studied development stages as sex is first detectable in zebrafish around 20-25 days post-fertilization (Aharon and Marlow, 2022), i.e. after all our analyses.

### Zebrafish staging and ethanol treatment

Eggs from random heterozygous crosses were collected and embryos were morphologically staged (Westerfield, 2007), sorted into sample groups of 100 and reared at 28.5°C to desired developmental time points. All groups were incubated in embryo medium (EM). At 10 hpf, EM was changed to either fresh EM or EM containing 1% ethanol (v/v). At 18 hpf, EM containing ethanol was washed out with three fresh changes of EM.

### Hybridization chain reaction, immunofluorescence and *in situ* hybridization

Embryos were collected at 36 hpf, dechorionated and fixed in 4% paraformaldehyde/PBS at 4°C. Hybridization chain reaction (HCR) protocol was as previously described (Ibarra-García-Padilla et al., 2021). HCR amplifiers and buffers were acquired from Molecular Instruments. HCR probes against *fgf8a* and *pdgfaa* were designed as previously described (Kuehn et al., 2022). Immunofluorescence was performed as previously described (Lovely et al., 2016). Primary antibodies were anti-GFP (1:200, sc-9996, Santa Cruz) (Lovely et al., 2016) and anti-mKO2 (1:200, PM051M, MBL) (Gillotay et al., 2020), secondary antibodies were Alexa Fluor 488 and Alexa Fluor 568 (1:500, A10042 and A21124, Invitrogen) (Lovely et al., 2016).

### Imaging and analysis of anterior pharyngeal endoderm shape, and Bmp signaling responses

Confocal images were taken using an Olympus FV1000 microscope and measured in FIJI (Schindelin et al., 2012). The anterior pharyngeal endoderm was defined as the medial endoderm anterior to the first pouch. General head area was defined as the product of the width and length of the embryo anterior to the first pouch. *BRE:mKO2* fluorescent intensity was measured in ImageJ by calculating the integrated density of the Bmp response in the pharyngeal arches as well as an average of three measures of mean background fluorescence (the fluorescence of the black background of the image. From these measures, the corrected total fluorescence (CTF) was calculated as: integrated density−(area×mean background fluorescence).

### Cartilage and bone staining

Zebrafish larvae fixed at 5 dpf were stained with Alcian Blue for cartilage and Alizarin Red for bone (Walker and Kimmel, 2007). Whole-mount, ventral view and brightfield images of the viscerocranium were taken on an Olympus BX53 compound microscope.

### Morphometric analysis

Morphometric analysis of larvae that had been stained using Alcian Blue and/or Alizarin Red was performed in TpsDig2 (https://www.sbmorphometrics.org) and MorphoJ (Klingenberg, 2011) (https://morphometrics.uk/MorphoJ_page.html). Landmarks were placed on the following joints: Meckel's cartilage midline joint, the joints between Meckel's and the palatoquadrate cartilage, the palatoquadrate and ceratohyal cartilage and at the end of the hyomandibular cartilage. Linear measures were analyzed using TpsDig2. Principal component analysis (PCA), Procrustes ANOVA and wireframe graphs of facial variation were generated using MorphoJ.

### Statistics

Meckel's cartilage area was analyzed with a one-way ANOVA with a Tukey's multiple comparisons test. Area measures of the anterior endoderm and/or head and linear measures and/or angles of both the anterior endoderm and the viscerocranium stained with Alcian Blue and/or Alizarin Red were analyzed using two-way ANOVA (type III) and a Tukey's multiple comparisons test in GraphPad Prism 9.5.1 (GraphPad Software Inc., La Jolla, CA, USA).

### Human studies

Human participants were recruited as part of the Collaborative Initiative on FASD (CIFASD) from (2007-2017) from sites in Atlanta, GA, Los Angeles, CA, Minneapolis, MN and San Diego, CA, USA (Mattson et al., 2010a,b). Institutional Review Board approval was obtained at each site. All participants and/or their parents/legal guardians provided written informed consent, and Institutional Review Board approval was obtained at each recruiting site. Children (aged 5-18 years) who had experienced heavy (>4 drinks/occasion at least once/week or >13 drinks/week) prenatal alcohol exposure (PAE) with or without a diagnosis of FAS were classified as alcohol exposed, those who had experienced minimal (<1 drink/week or <2 drinks/occasion) or no PAE were classified as not alcohol exposed (Mattson et al., 2010a,b). 3D images of patients were obtained of most participants at the time of the dysmorphology exam by using static stereophotogrammetric camera systems capable of capturing a 180-degree image of facial surface geometry. Images were annotated with a sparse set of 20 reliable anthropometric landmarks (Fig. S7A). We performed a Procrustes alignment on the landmarks of each face, aligning them to a template face using a similarity transform, which was applied to each 3D surface to normalize size to a uniform scale. Dense surface models (DSMs) have previously been used for the assessment of subtle facial dysmorphia across the FASD spectrum (Suttie et al., 2013, 2017), and provide a method to assess surface-based differences of 3D structures, and compare groups of individuals to assess mean differences. A DSM containing 303 individuals was constructed from the uniformly scaled images to produce shape-only morphometric models. DSMs allow us to compute facial signatures, which represent normalized differences between groups, and visualize using a heatmap representation group or individual differences (Hammond et al., 2012). To evaluate micrognathia, we computed the mandible volume, which was outlined and validated against CT images as described by Basart et al. (2018). This technique estimated the volume of a trapezoid formed by four specific points on the size-normalized face: the left and right lower otobasion inferius (lowest points of the ears), gnathion (tip of the mandible) and lower lip vermillion center (Fig. S7B).

Genome-wide association study (GWAS) data were genotyped on the OmniExpress genome array (Illumina, San Diego, CA, USA) and on the Multi-Ethnic Genotyping Array at the Johns Hopkins Genetic Resources Core Facility (Baltimore, MD, USA; Dou et al., 2018). Following a previously published GWAS cleaning pipeline (Schwantes–An et al., 2021), the two datasets were cleaned for sample and variant call rates, Hardy–Weinberg equilibrium (HWE), sample identity using genetic − calculated from single nucleotide polymorphisms (SNPs) on X and Y chromosomes − and self-reported sex, sample relatedness and genetic ancestry. The two cleaned datasets were imputed separately using the Michigan Imputation Server (Das et al., 2016) to 1000-Genomes Phase 3, b37 reference panel (Fairley et al., 2020) and then combined. The final dataset consisted of 4,000,362 genotyped and imputed SNPs with minor allele frequency (MAF) ≥0.01, genotype rate ≥0.99, and Hardy–Weinberg equilibrium *P*≥0.000001. Principal components analysis (PCA) was performed using SNPRelate (Zheng et al., 2012) with genotype data from autosomes. Individuals in the 1000-Genomes database (Fairley et al., 2020) were included as reference samples for clustering individuals based on genetic ancestry similarities. Individuals with genotype data were grouped with European Ancestry (EA; *n*=222), African Ancestry (AA; *n*=103) or other (*n*=44) ancestry samples from 1000 Genomes Project using the first three principal components. Specific for the top SNP in *BMPR1B*, the mandible volume was available for 184 EA and 135 AA individuals, which were included in our analyses.

Analyses were performed separately in the EA and AA groups using R (version 4.2.0; R Foundation for Statistical Computing; https://www.r-project.org/foundation/) and PLINK v2.00a3 64-bit (8 Jun 2021). Association of SNP genotype with mandible volume was assessed using an additive generalized linear model with sex, age at time of 3D image, the first ten genetic principal components, genotype, PAE and additive genotype by PAE interaction. As the effect of interest was the association of genotype with mandible volume with and without the presence of PAE, the *P*-value from the generalized linear model for the genotype by PAE interaction is reported. Variants within 25 kb of the three genes of interest [*BMP2* (6 SNPs), *BMP4* (8 SNPs), *BMPR1B* (602 SNPs)] were evaluated in the EA group to identify significant SNPs. As there is a wide range of correlation between SNPs within a gene, we corrected for the number of independent SNPs as a proxy for the number of independent tests within each gene, as follows. We first utilized *r^2^* values obtained from LDlink (Machiela and Chanock, 2015) between all tested SNP pairs in each gene. Since significant results were revealed only in the EA sample, the EUR ancestry group (The 1000 Genomes Project Consortium. A global reference for human genetic variation, 2015) was utilized. We conservatively assumed that variants not available in the reference data were not in linkage disequilibrium (LD) with all other SNPs. Then matSpD (Nyholt, 2004) was performed on the matrix of *r^2^* values, with the Li and Ji correction applied (Li and Ji, 2005), to estimate the number of effective independent tests for each gene. This yielded five independent tests for *BMP2* (adjusted alpha=0.01), six tests for *BMP4* (adjusted alpha=0.0083), and 103 tests for *BMPR1B* (adjusted alpha=0.00048). Due to the small sample size and the inability to replicate these results, we did not follow up on any results that did not meet the adjusted alpha for significance. To further explore the genotype by PAE interaction, we employed the non-parametric Dwass−Steel−Critchlow−Fligner method (SAS v 9.4) to estimate the pairwise comparisons of genotype×PAE interaction. A 3D image was not available for all individuals with genotype data.

## Supplementary Material

10.1242/dmm.052223_sup1 Supplementary information
