# Supplementary material for: Mutations in the bone morphogenetic protein signaling pathway sensitize zebrafish and humans to ethanol-induced jaw malformations
Source: Dis Model Mech. 2025 Apr 8;18(4):dmm052223. doi: 10.1242/dmm.052223 (PMC12010914; doi:10.1242/dmm.052223)
Supplement: Supplementary information [file dmm-18-052223-s1.pdf]

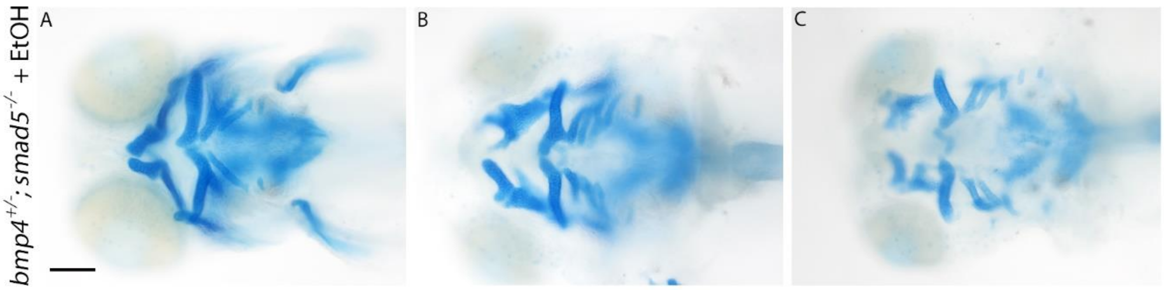


**Fig. S1. A wide spectrum of viscerocranial malformations was observed in ethanol-treated *bmp4*$^{+/-}$*;smad5*$^{-/-}$ larvae.** (**A-C**) Whole-mount images of viscerocranium at 5 dpf larvae. Cartilage is blue and bone is red (Ventral views, anterior to the left, scale bar: 100 µm). (**A-C**) Ethanol exposure on *bmp4*$^{+/-}$ *;smad5*$^{-/-}$ larvae range from stereotypical *smad5* mutant phenotypes to severe ethanol-induced phenotypes to the viscerocranium.

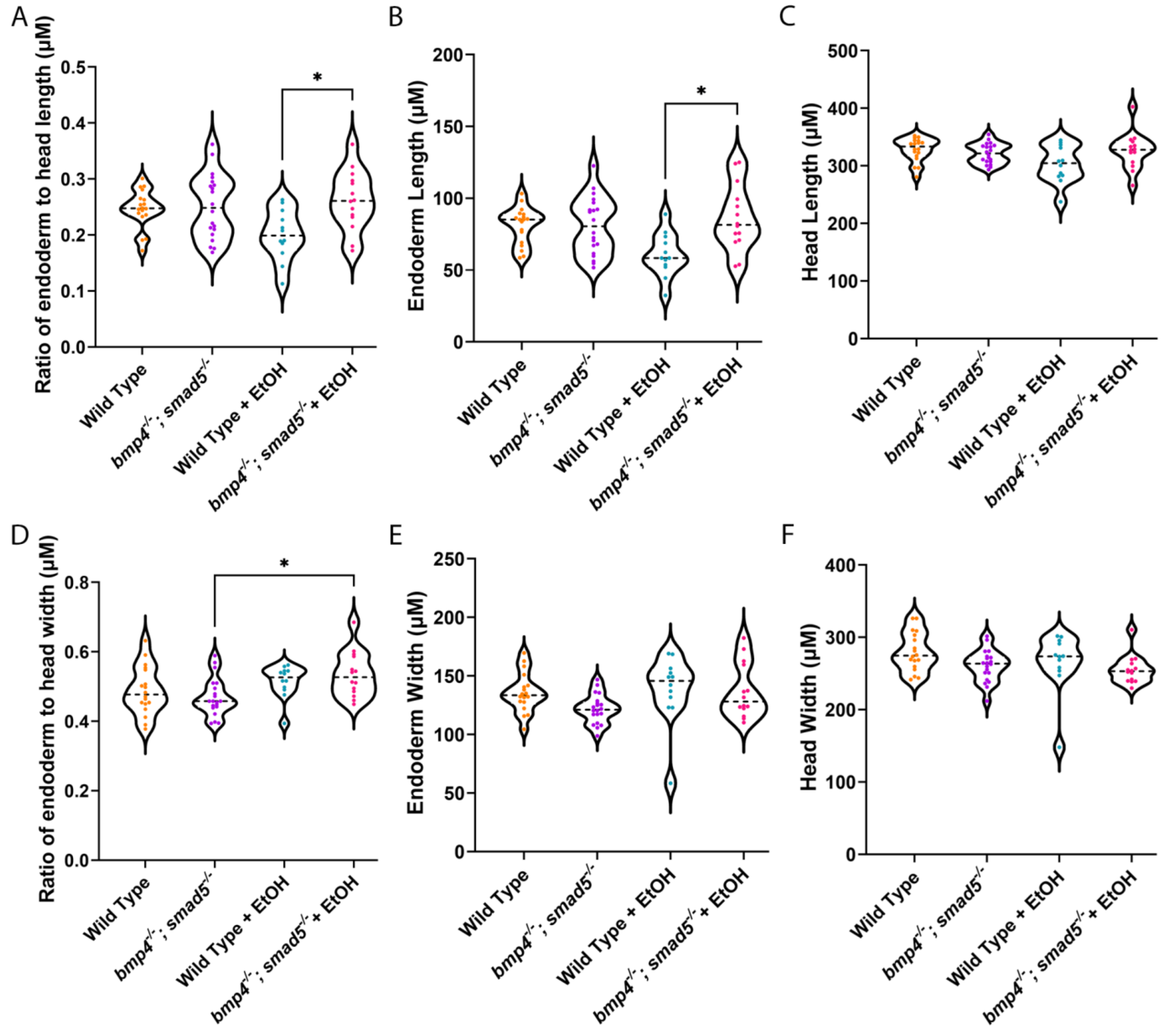


**Fig. S2. Ethanol exposure alters size of the anterior pharyngeal endoderm in *bmp4*$^{-/-}$ *;smad5*$^{-/-}$ embryos.** (**A-F**) Length and width measures of the anterior pharyngeal endoderm. (**A-C**) Ethanol exposure decreased endoderm length but not head length in Wild Type embryos but significantly increased endoderm length ethanol-treated *bmp4*$^{-/-}$*;smad5*$^{-/-}$ embryos. (**D-F**) Ethanol-treatment significantly increased in the ratio of endoderm width to head width in *bmp4*$^{-/-}$*;smad5*$^{-/-}$ embryos, though there was a trend in the increase in ethanol-treated Wild Type embryos as well, but not in head width (individual graph statistics in Supplemental Table 5).

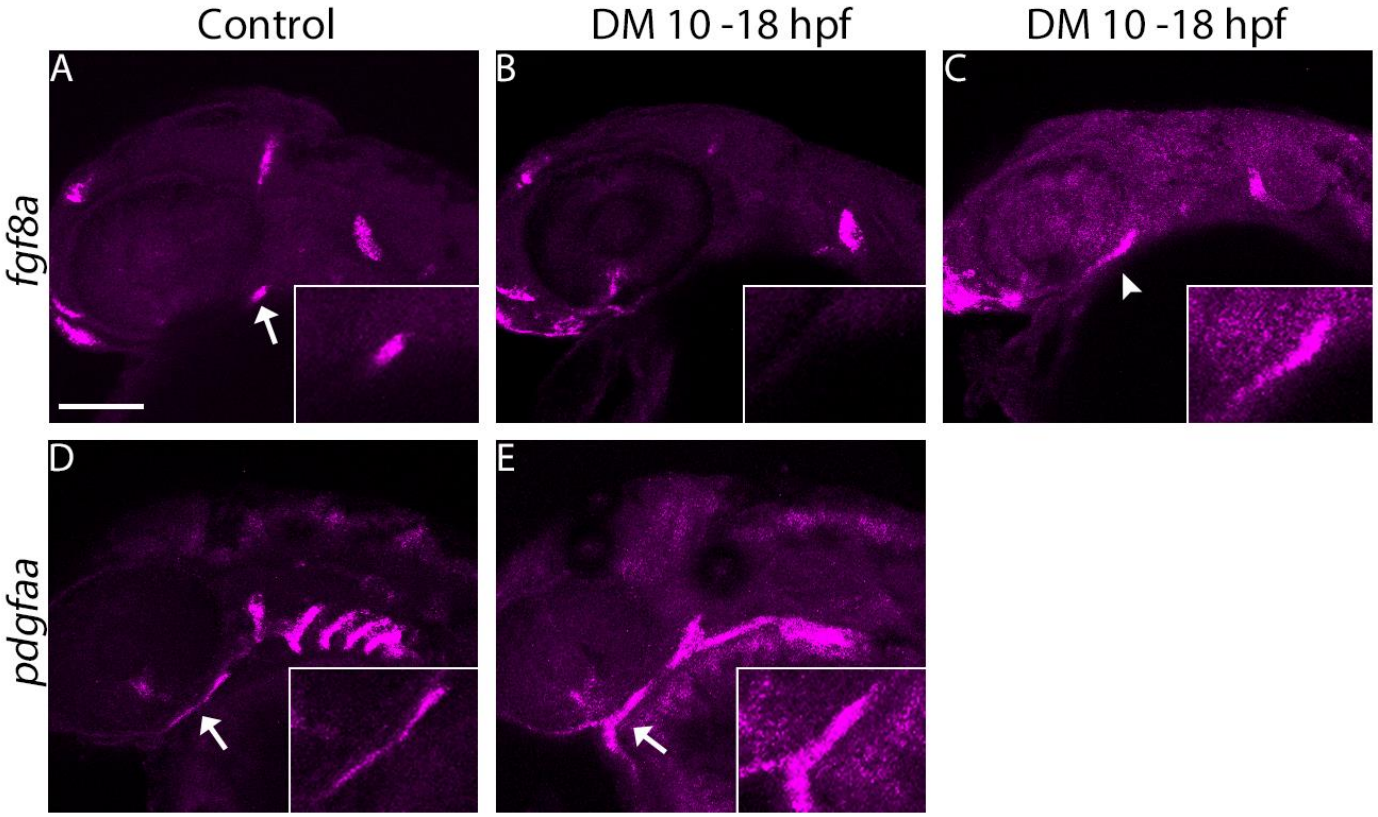


**Fig. S3. Blocking Bmp signaling with Dorsomorphin inhibitor (DM) disrupts the expression of *fgf8a* in the oral ectoderm.** (**A-F**) Whole-mount images of untreated and DM-treated embryos labeling *fgf8a* and *pdgfaa* gene expression at 36 hpf (lateral views, anterior to the left, scale bar: 100 µm). (**A&D**) Normal expression of *fgf8a* and *pdgfaa* in the oral ectoderm of untreated embryos. (**B, C, E**) Expression of *fgf8a* is lost in DM-treated embryos, while *pdgfaa* are expressed normally (n = 20 embryos per group).

Disease Models & Mechanisms • Supplementary information

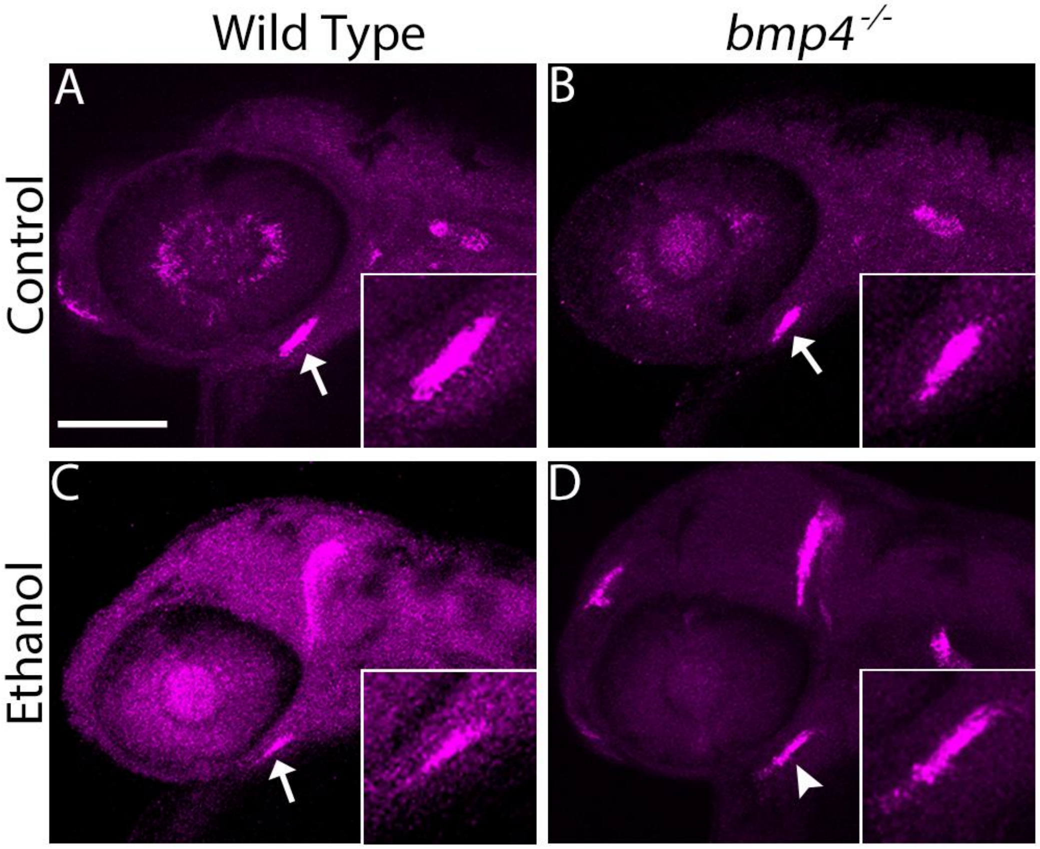


**Fig. S4. Ethanol exposure changes shape of oral ectoderm expression domain in *bmp4*$^{-/-}$ embryos.** (**A-D**) Whole-mount, confocal images of *bmp4* embryos fluorescently labeling *fgf8a* gene expression at 36 hpf (lateral views, anterior to the left, scale bar: 100 µm). (**A-C**) Arrows show normal expression of *fgf8a* in the oral ectoderm of untreated wild type and *bmp4*$^{-/-}$ embryos as well as ethanol-treated wild type embryos. (**D**) Arrowhead shows that domain of *fgf8a* expression in ethanol-treated *bmp4*$^{-/-}$ embryos is subtly expanded anteriorly (n = 5 embryos per group).

Disease Models & Mechanisms • Supplementary information

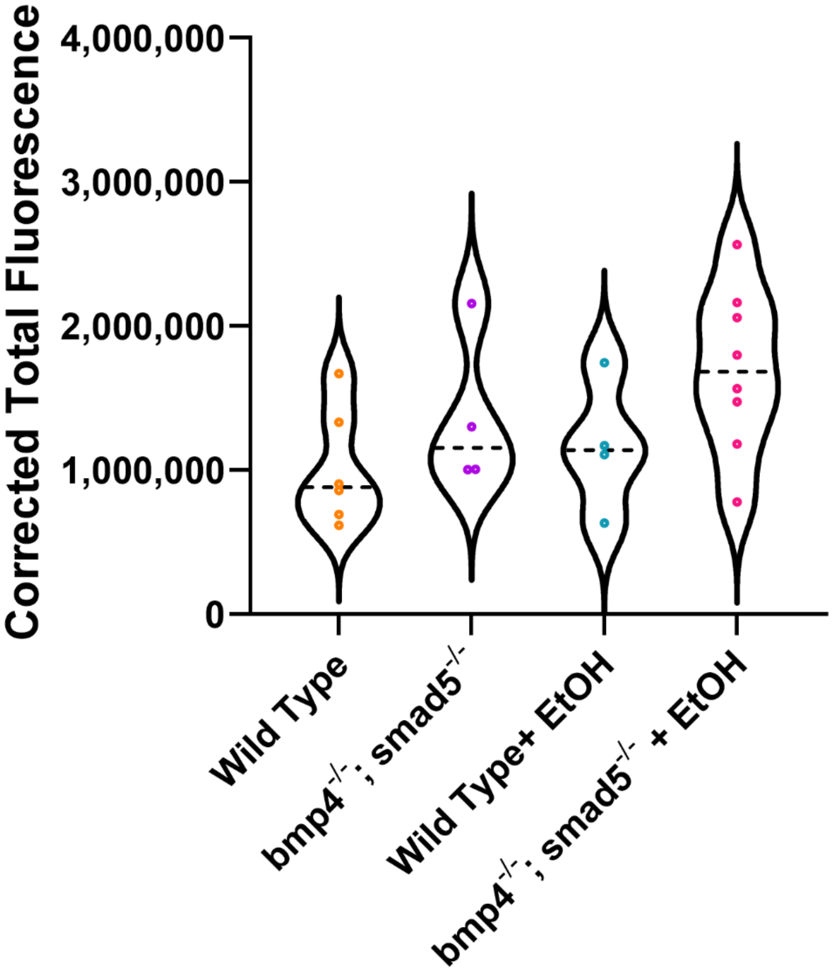


**Fig. S5. Ethanol exposure does not lead to decreases in Bmp signaling responses**.

Corrected Total Fluorescence was calculated from *BRE:mKO2* fluorescence. We observed no change in BRE signaling responses in *bmp4$^{-/-}$;smad5$^{-/-}$* embryos or due to ethanol. (Embryos per group, wild type, n = 5; *bmp4$^{-/-}$ ;smad5$^{-/-}$* , n = 4; wild type + Etoh, n = 4; *bmp4$^{-/-}$;smad5$^{-/-}$*, n = 8).

Disease Models & Mechanisms • Supplementary information

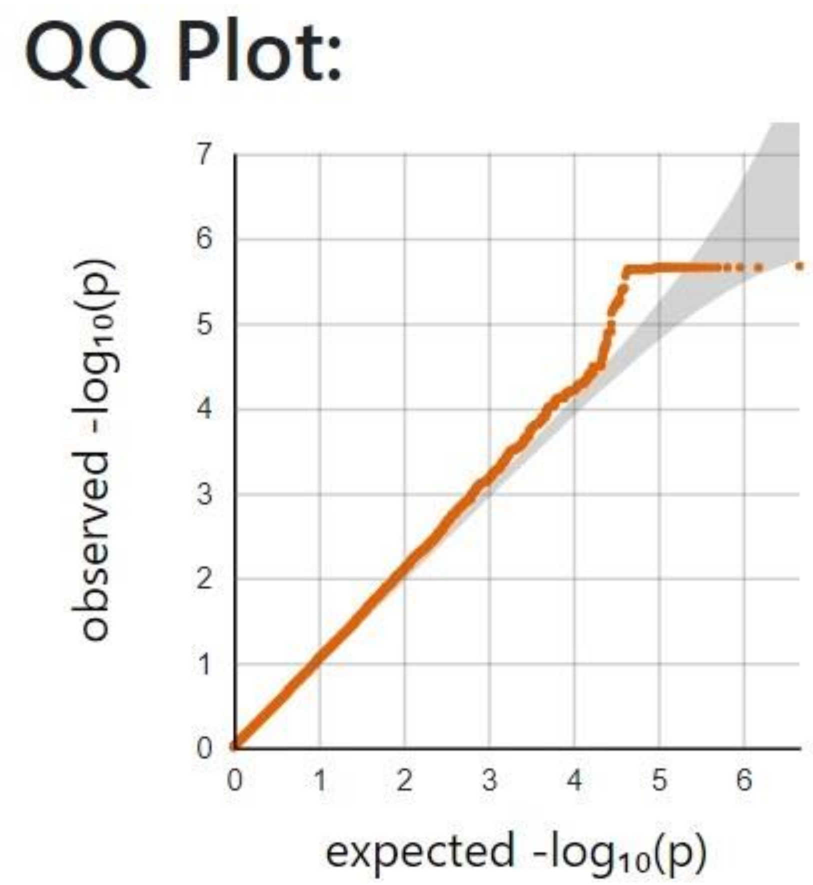


**Fig. S6. Quantile-quantile (QQ) plot for the genotype x prenatal alcohol exposure interaction p-values from the genome-wide association study of mandible volume.**

Disease Models & Mechanisms • Supplementary information

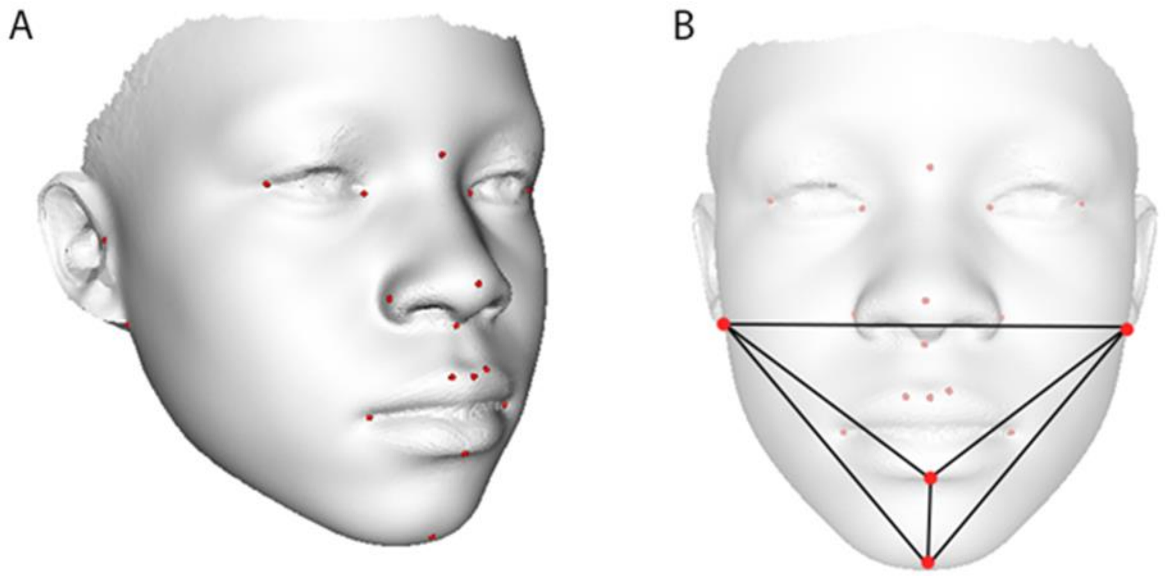


**Fig. S7. Visual representation of the 20 landmark locations used to induce the dense surface correspondence. (A-B)** left and right endocanthion, exocanthion, tragion, otobasion inferius, crista philtrum, cheilion, and alare; nasion, pronasale, subnasale, labiale superius, labiale inferius, and gnathion. **(B)** The landmarks labiuminferius, gnathion, and otobasion inferior define the tetrahedron (1,2,3,4). The volume of the tetrahedron is calculated as (1/6)det (axayaz1; bxbybz1; cxcycz1; dxdydz1).

Disease Models & Mechanisms • Supplementary information

**Table S1. Timing of *bmp4*-ethanol penetrance.** Percent of jaw malformations and jaw loss per exposure start window in ethanol-treated *bmp4* mutant embryos generated from random heterozygous crosses.

**Supplemental Table 1. Timing of *bmp4*-ethanol penetrance**

| Experiments | Untreated Embryos | EtOH-treated Embryos | Jaw Malformations | Percent | Jaw Loss | Percent |
|---|---|---|---|---|---|---|
| Control | 97 | | 0 | 0.0% | 0 | 0.0% |
| E10 | | 174 | 42 | 24.1% | 6 | 3.4% |
| E14 | | 193 | 23 | 11.9% | 7 | 3.6% |
| E18 | | 192 | 18 | 9.4% | 1 | 0.5% |

**Table S2. Dose response of *bmp4*-ethanol penetrance.** Percent of jaw malformations and jaw loss per dose at starting at 24 hpf in ethanol-treated *bmp4* mutant embryos generated from random heterozygous crosses.

**Supplemental Table 2. Dose response of *bmp4*-ethanol penetrance after 24 hpf**

| Experiments | Untreated Embryos | EtOH-treated Embryos | Jaw Malformations | Percent | Jaw Loss | Percent |
|---|---|---|---|---|---|---|
| Control | 138 | | 0 | 0.0% | 0 | 0.0% |
| E 1% | | 165 | 2 | 1.2% | 0 | 0.0% |
| E 1.1% | | 159 | 2 | 1.3% | 0 | 0.0% |
| E 1.2% | | 165 | 2 | 1.2% | 0 | 0.0% |
| E 1.3% | | 159 | 2 | 1.3% | 0 | 0.0% |

Disease Models & Mechanisms • Supplementary information

**Table S3. Statistical analyses of linear facial measures in Figure 2.** Alcian/alizarin-stained viscerocraniums were analyzed with a two-way ANOVA (type III). F-statistic and P-value for each analysis are shown. A Tukey's Multiple Comparisons Test for each comparison are shown.

**Supplemental Table 3. Length Measure Statistics for Figure 2**

| **Length at MC-Ch joints / Head Length** | | |
|---|---|---|
| **ANOVA** | F = 7.689 | P < 0.0001 |
| **Tukey's Comparisons** | | |
| Wild type vs *bmp4*$^{-/-}$ | p = 0.05 | |
| Wild type vs wild type + EtOH | p = 0.9966 | |
| *Wild type* vs *bmp4*$^{-/-}$ + EtOH | p = .1067 | |
| *bmp4*$^{-/-}$ vs wild type + EtOH | p = 0.0292 | |
| *bmp4*$^{-/-}$ vs *bmp4*$^{-/-}$ + EtOH | p < 0.0001 | |
| *Wild type* + EtOH vs *bmp4*$^{-/-}$ + EtOH | p = 0.1838 | |

| **Width at MC/PQ joint / Head Length** | | |
|---|---|---|
| **ANOVA** | F = 9.747 | P < 0.0001 |
| **Tukey's Comparisons** | | |
| Wild type vs *bmp4*$^{-/-}$ | p = 0.002 | |
| Wild type vs wild type + EtOH | p = 0.3758 | |
| *Wild type* vs *bmp4*$^{-/-}$ + EtOH | p = 0.4716 | |
| *bmp4*$^{-/-}$ vs wild type + EtOH | p < 0.0001 | |
| *bmp4*$^{-/-}$ vs *bmp4*$^{-/-}$ + EtOH | p = .1074 | |
| *Wild type* + EtOH vs *bmp4*$^{-/-}$ + EtOH | p = 0.0145 | |

| **Width at Ch-PQ joint / Head Length** | | |
|---|---|---|
| **ANOVA** | F = 14.96 | P < 0.0001 |
| **Tukey's Comparisons** | | |
| Wild type vs *bmp4*$^{-/-}$ | p = 0.0033 | |
| Wild type vs wild type + EtOH | p = 0.0073 | |
| *Wild type* vs *bmp4*$^{-/-}$ + EtOH | p = 0.957 | |
| *bmp4*$^{-/-}$ vs wild type + EtOH | p < 0.0001 | |
| *bmp4*$^{-/-}$ vs *bmp4*$^{-/-}$ + EtOH | p = 0.0005 | |
| *Wild type* + EtOH vs *bmp4*$^{-/-}$ + EtOH | p = 0.0305 | |

| **Angle at MC / Head Length** | | |
|---|---|---|
| **ANOVA** | F = 15.02 | P < 0.0001 |
| Tukey's Comparisons | | |
| Wild type vs *bmp4*$^{-/-}$ | p = 0.3508 | |
| Wild type vs wild type + EtOH | p = 0.1032 | |
| *Wild type* vs *bmp4*$^{-/-}$ + EtOH | p < 0.0001 | |
| *bmp4*$^{-/-}$ vs wild type + EtOH | p = 0.9371 | |
| *bmp4*$^{-/-}$ vs *bmp4*$^{-/-}$ + EtOH | p < 0.0001 | |
| *Wild type* + EtOH vs *bmp4*$^{-/-}$ + EtOH | p = 0.0005 | |

| **Angle at Ch / Head Length** | | |
|---|---|---|
| **ANOVA** | F = 14.89 | P < 0.0001 |
| Tukey's Comparisons | | |
| Wild type vs *bmp4*$^{-/-}$ | p = 0.0674 | |
| Wild type vs wild type + EtOH | p = 0.0048 | |
| *Wild type* vs *bmp4*$^{-/-}$ + EtOH | p < 0.0001 | |
| *bmp4*$^{-/-}$ vs wild type + EtOH | p = 0.8394 | |
| *bmp4*$^{-/-}$ vs *bmp4*$^{-/-}$ + EtOH | p = 0.001 | |
| *Wild type* + EtOH vs *bmp4*$^{-/-}$ + EtOH | p = 0.0128 | |

| **Head Length** | | |
|---|---|---|
| **ANOVA** | F = 18.46 | P < 0.0001 |
| Tukey's Comparisons | | |
| Wild type vs *bmp4*$^{-/-}$ | p = 0.9725 | |
| Wild type vs wild type + EtOH | p = 0.011 | |
| *Wild type* vs *bmp4*$^{-/-}$ + EtOH | p < 0.0001 | |
| *bmp4*$^{-/-}$ vs wild type + EtOH | p = 0.0477 | |
| *bmp4*$^{-/-}$ vs *bmp4*$^{-/-}$ + EtOH | p < 0.0001 | |
| *Wild type* + EtOH vs *bmp4*$^{-/-}$ + EtOH | p = 0.0047 | |

| **Perimeter Length of MC** | | |
|---|---|---|
| **ANOVA** | F = 3.525 | P = 0.0163 |
| **Tukey's Comparisons** | | |
| Wild type vs *bmp4*$^{-/-}$ | p = 0.9917 | |
| Wild type vs wild type + EtOH | p = 0.9748 | |
| *Wild type* vs *bmp4*$^{-/-}$ + EtOH | p = 0.0223 | |
| *bmp4*$^{-/-}$ vs wild type + EtOH | p = 0.9993 | |
| *bmp4*$^{-/-}$ vs *bmp4*$^{-/-}$ + EtOH | p = 0.067 | |
| *Wild type* + EtOH vs *bmp4*$^{-/-}$ + EtOH | p = 0.0786 | |

| **Perimeter Length of PQ** | | |
|---|---|---|
| ANOVA | F = 36.27 | P < 0.0001 |
| **Tukey's Comparisons** | | |
| Wild type vs *bmp4*$^{-/-}$ | p = 0.4074 | |
| Wild type vs wild type + EtOH | p < 0.0001 | |
| *Wild type* vs *bmp4*$^{-/-}$ + EtOH | p < 0.0001 | |
| *bmp4*$^{-/-}$ vs wild type + EtOH | p = 0.0002 | |
| *bmp4*$^{-/-}$ vs *bmp4*$^{-/-}$ + EtOH | p < 0.0001 | |
| *Wild type* + EtOH vs *bmp4*$^{-/-}$ + EtOH | p = 0.0093 | |

**Table S4. Statistical analyses of endoderm/head area measures in Figure 4.** Measures of anterior endoderm/head area were analyzed with a two-way ANOVA (type III). F-statistic and P-value for each analysis are shown. A Tukey's Multiple Comparisons Test for each comparison are shown.

**Supplemental Table 4. Statistics for Figure 4**

| **Ratio of Endoderm to Head Area** | | **Endoderm Area** | | **Head Area (Calculated)** | |
|---|---|---|---|---|---|
| **ANOVA** | F = 5.919 P = 0.0019 | **ANOVA** | F = 2.801 P = 0.0518 | **ANOVA** | F = 1.958 P = 0.1353 |
| **Tukey's Comparisons** | | **Tukey's Comparisons** | | **Tukey's Comparisons** | |
| Wild type vs *bmp4*$^{-/-}$ *;smad5*$^{+/+}$ | p = 0.9989 | Wild type vs *bmp4*$^{-/-}$ *;smad5*$^{+/+}$ | p = 0.8751 | Wild type vs *bmp4*$^{-/-}$ *;smad5*$^{+/+}$ | p = 0.3505 |
| Wild type vs wild type + EtOH | p = 0.8727 | Wild type vs wild type + EtOH | p = 0.8751 | Wild type vs wild type + EtOH | p = 0.1492 |
| Wild type vs *bmp4*$^{-/-}$ *;smad5*$^{-/-}$ + EtOH | p = 0.0127 | Wild type vs *bmp4*$^{-/-}$ *;smad5*$^{-/-}$ + EtOH | p = 0.8751 | Wild type vs *bmp4*$^{-/-}$ *;smad5*$^{-/-}$ + EtOH | p = 0.2876 |
| *bmp4*$^{-/-}$;smad5$^{-/-}$ vs wild type + EtOH | p = 0.9211 | *bmp4*$^{-/-}$;smad5$^{-/-}$ vs wild type + EtOH | p = 0.8751 | *bmp4*$^{-/-}$;smad5$^{-/-}$ vs wild type + EtOH | p = 0.8835 |
| *bmp4*$^{-/-}$;smad5$^{-/-}$ vs *bmp4*$^{-/-}$*;smad5*$^{-/-}$ + EtOH | p = 0.0089 | *bmp4*$^{-/-}$;smad5$^{-/-}$ vs *bmp4*$^{-/-}$*;smad5*$^{-/-}$ + EtOH | p = 0.1277 | *bmp4*$^{-/-}$;smad5$^{-/-}$ vs *bmp4*$^{-/-}$*;smad5*$^{-/-}$ + EtOH | p = 0.992 |
| *bmp4*$^{-/-}$;smad5$^{-/-}$ + EtOH vs *bmp4*$^{-/-}$ *;smad5*$^{-/-}$ + EtOH | p = 0.0033 | *bmp4*$^{-/-}$;smad5$^{-/-}$ + EtOH vs *bmp4*$^{-/-}$ *;smad5*$^{-/-}$ + EtOH | p = 0.0494 | *bmp4*$^{-/-}$;smad5$^{-/-}$ + EtOH vs *bmp4*$^{-/-}$ *;smad5*$^{-/-}$ + EtOH | p = 0.9686 |

**Table S5. Statistical analyses of linear anterior endoderm/head measures in Fig. S2.**

Linear measures of anterior endoderm/head were analyzed with a two-way ANOVA (type III). F-statistic and P-value for each analysis are shown. A Tukey's Multiple Comparisons Test for each comparison are shown.

**Supplemental Table 5. Statistics for Supplemental Figure 2**

| **Ratio of Endoderm to Head Length** | | **Endoderm Length** | | **Head Length** | |
|---|---|---|---|---|---|
| **ANOVA** | F = 2.930 P = 0.0448 | **ANOVA** | F = 3.811 P = 0.0169 | **ANOVA** | F = 2.309 P = 0.0906 |
| **Tukey's Comparisons** | | **Tukey's Comparisons** | | **Tukey's Comparisons** | |
| Wild type vs *bmp4*$^{-/-}$*;smad5*$^{+/+}$ | p = 0.9989 | Wild type vs *bmp4*$^{-/-}$*;smad5*$^{+/+}$ | p = 0.8751 | Wild type vs *bmp4*$^{-/-}$*;smad5*$^{+/+}$ | p = 0.3505 |
| Wild type vs wild type + EtOH | p = 0.8727 | Wild type vs wild type + EtOH | p = 0.8751 | Wild type vs wild type + EtOH | p = 0.1492 |
| Wild type vs *bmp4*$^{-/-}$*;smad5*$^{-/-}$ + EtOH | p = 0.0127 | Wild type vs *bmp4*$^{-/-}$*;smad5*$^{-/-}$ + EtOH | p = 0.8751 | Wild type vs *bmp4*$^{-/-}$*;smad5*$^{-/-}$ + EtOH | p = 0.2876 |
| *bmp4*$^{-/-}$;smad5$^{-/-}$ vs wild type + EtOH | p = 0.9211 | *bmp4*$^{-/-}$;smad5$^{-/-}$ vs wild type + EtOH | p = 0.8751 | *bmp4*$^{-/-}$;smad5$^{-/-}$ vs wild type + EtOH | p = 0.8835 |
| *bmp4*$^{-/-}$;smad5$^{-/-}$ vs *bmp4*$^{-/-}$*;smad5*$^{-/-}$ + EtOH | p = 0.0089 | *bmp4*$^{-/-}$;smad5$^{-/-}$ vs *bmp4*$^{-/-}$*;smad5*$^{-/-}$ + EtOH | p = 0.1277 | *bmp4*$^{-/-}$;smad5$^{-/-}$ vs *bmp4*$^{-/-}$*;smad5*$^{-/-}$ + EtOH | p = 0.992 |
| *bmp4*$^{-/-}$;smad5$^{-/-}$ + EtOH vs *bmp4*$^{-/-}$*;smad5*$^{-/-}$ + EtOH | p = 0.0033 | *bmp4*$^{-/-}$;smad5$^{-/-}$ + EtOH vs *bmp4*$^{-/-}$*;smad5*$^{-/-}$ + EtOH | p = 0.0494 | *bmp4*$^{-/-}$;smad5$^{-/-}$ + EtOH vs *bmp4*$^{-/-}$*;smad5*$^{-/-}$ + EtOH | p = 0.9686 |

| **Ratio of Endoderm to Head Width** | | **Endoderm Width** | | **Head Width** | |
|---|---|---|---|---|---|
| **ANOVA** | F = 3.063 P = 0.0386 | **ANOVA** | F = 1.869 P = 0.1499 | **ANOVA** | F = 2.691 P = 0.0587 |
| **Tukey's Comparisons** | | **Tukey's Comparisons** | | **Tukey's Comparisons** | |
| Wild type vs *bmp4*$^{-/-}$*;smad5*$^{+/+}$ | p = 0.8133 | Wild type vs *bmp4*$^{-/-}$*;smad5*$^{+/+}$ | p = 0.2316 | Wild type vs *bmp4*$^{-/-}$*;smad5*$^{+/+}$ | p = 0.1886 |
| Wild type vs wild type + EtOH | p = 0.652 | Wild type vs wild type + EtOH | p = 0.9983 | Wild type vs wild type + EtOH | p = 0.3263 |
| Wild type vs *bmp4*$^{-/-}$*;smad5*$^{-/-}$ + EtOH | p = 0.1846 | Wild type vs *bmp4*$^{-/-}$*;smad5*$^{-/-}$ + EtOH | p = 0.9993 | Wild type vs *bmp4*$^{-/-}$*;smad5*$^{-/-}$ + EtOH | p = 0.0523 |
| *bmp4*$^{-/-}$;smad5$^{-/-}$ vs wild type + EtOH | p = 0.2343 | *bmp4*$^{-/-}$;smad5$^{-/-}$ vs wild type + EtOH | p = 0.277 | *bmp4*$^{-/-}$;smad5$^{-/-}$ vs wild type + EtOH | p > 0.9999 |
| *bmp4*$^{-/-}$;smad5$^{-/-}$ vs *bmp4*$^{-/-}$*;smad5*$^{-/-}$ + EtOH | p = 0.0324 | *bmp4*$^{-/-}$;smad5$^{-/-}$ vs *bmp4*$^{-/-}$*;smad5*$^{-/-}$ + EtOH | p = 0.2553 | *bmp4*$^{-/-}$;smad5$^{-/-}$ vs *bmp4*$^{-/-}$*;smad5*$^{-/-}$ + EtOH | p = 0.8529 |
| *bmp4*$^{-/-}$;smad5$^{-/-}$ + EtOH vs *bmp4*$^{-/-}$*;smad5*$^{-/-}$ + EtOH | p = 0.8716 | *bmp4*$^{-/-}$;smad5$^{-/-}$ + EtOH vs *bmp4*$^{-/-}$*;smad5*$^{-/-}$ + EtOH | p > 0.9999 | *bmp4*$^{-/-}$;smad5$^{-/-}$ + EtOH vs *bmp4*$^{-/-}$*;smad5*$^{-/-}$ + EtOH | p = 0.866 |

**Table S6. Calculation of Corrected Total Fluorescence for Figures 6 & S5.** Pharyngeal area of *BRE:mKO2* fluorescent intensity was quantified using Image J. Corrected Total Fluorescence (unitless) was calculated from Integrated Density – (Area of fluorescence x mean background fluorescence). Integrated density is the Sum of all pixels within the area of fluorescent measurement. Mean background fluorescence was the average of three independent measures.

Disease Models & Mechanisms • Supplementary information

**Supplemental Table 6. Calculation of Bmp Signaling Response for Figures 6 and S5**

**Wild type**

| | Embryo 1 | | Embryo 2 | | Embryo 3 | | Embryo 4 | | Embryo 5 | | Embryo 6 | |
|---|---|---|---|---|---|---|---|---|---|---|---|---|
| | Area[1] | IntDent[2] | Area | IntDent | Area | IntDent | Area | IntDent | Area | IntDent | Area | IntDent |
| Bmp Response Fluorescence | 2733.42 | 719227.68 | 3953.59 | 1699423.2 | 4806.63 | 960200.14 | 1920.49 | 636671 | 2532.89 | 903406.13 | 3173.05 | 1378586.06 |
| Mean Background Fluorescence[3] | | 10.12 | | 7.35 | | 11.80 | | 10.66 | | 17.29 | | 14.65 |
| Corrected Total Fluorescence[4] | | 691556.36 | | 1670351.13 | | 903465.88 | | 616204.98 | | 859620.90 | | 1332111.45 |

***bmp4$^{-/-}$;smad5$^{-/-}$***

| | Embryo 1 | | Embryo 2 | | Embryo 3 | | Embryo 4 | |
|---|---|---|---|---|---|---|---|---|
| | Area | IntDent | Area | IntDent | Area | IntDent | Area | IntDent |
| Bmp Response Fluorescence | 2776.62 | 1040638.57 | 2758.1 | 1338034.92 | 5244.72 | 1079115.85 | 5696.69 | 2215054.56 |
| Mean Background Fluorescence[3] | | 12.25 | | 13.77 | | 14.62 | | 10.39 |
| Corrected Total Fluorescence[4] | | 1006624.98 | | 1300046.69 | | 1002438.04 | | 2155846.96 |

**Wild type + Etoh**

| | Embryo 1 | | Embryo 2 | | Embryo 3 | | Embryo 4 | |
|---|---|---|---|---|---|---|---|---|
| | Area | IntDent | Area | IntDent | Area | IntDent | Area | IntDent |
| Bmp Response Fluorescence | 4786.58 | 1238510.01 | 2295.34 | 654548.45 | 4205.03 | 1812270.79 | 2542.15 | 1151769.7 |
| Mean Background Fluorescence | | 14.38 | | 9.92 | | 15.92 | | 16.99 |
| Corrected Total Fluorescence | | 1169694.94 | | 631771.03 | | 1745340.73 | | 1108570.10 |

***bmp4$^{-/-}$;smad5$^{-/-}$* + Etoh**

| | Embryo 1 | | Embryo 2 | | Embryo 3 | | Embryo 4 | | Embryo 5 | | Embryo 6 | | Embryo 7 | | Embryo 8 | |
|---|---|---|---|---|---|---|---|---|---|---|---|---|---|---|---|---|
| | Area | IntDent | Area | IntDent | Area | IntDent | Area | IntDent | Area | IntDent | Area | IntDent | Area | IntDent | Area | IntDent |
| Bmp Response Fluorescence | 3333.48 | 1519555.03 | 5056.52 | 1248038.26 | 2363.21 | 812945.68 | 12806.37 | 1943733.14 | 6509.62 | 2634085.11 | 4428.7 | 1614516.1 | 6714.78 | 2117435.12 | 4326.89 | 2239574.08 |
| Mean Background Fluorescence | | 13.44 | | 13.41 | | 14.51 | | 11.23 | | 10.66 | | 11.02 | | 8.66 | | 17.58 |
| Corrected Total Fluorescence | | 1474741.95 | | 1180213.47 | | 778647.63 | | 1799960.29 | | 2564670.86 | | 1565711.83 | | 2059262.74 | | 2163507.35 |

[1]Area = Area of measured Bmp-dependent GFP Fluorescence
[2]IntDent (Integrated Density) = Sum of all pixels within the area of measured Bmp-dependent GFP Fluoresence
[3]Mean background fluorescence of image is an average of three independent measures
[4]Corrected Total Fluorescence = Integrated Density – (Area X Mean background fluorescence)
